# A single-residue deletion in H9N2 hemagglutinin uncouples viral burden from disease severity

**DOI:** 10.1371/journal.ppat.1014776

**Published:** 2026-11-05

**Authors:** Yixue Sun, Yanting Zhu, Pengju Zhang, Shouzhi Sheng, Lili Gai, Siqi Li, Fangyu Zhang, Peige Cao, Ao He, Weixia Wang, Xintao Li, Yanlong Cong

**Affiliations:** 1 State Key Laboratory for Diagnosis and Treatment of Severe Zoonotic Infectious Diseases, Key Laboratory for Zoonosis Research of the Ministry of Education, and College of Veterinary Medicine, Jilin University, Changchun, China; 2 Jilin Research and Development Center of Biomedical Engineering, Changchun University, Changchun, China; 3 Jilin Academy of Agricultural Sciences (Northeast Innovation Center for Agricultural Science and Technology in China), Changchun, China; 4 Institute of Animal Biotechnology, Jilin Academy of Agricultural Sciences, Changchun, China; University of North Carolina at Chapel Hill, UNITED STATES OF AMERICA

## Abstract

H9N2 avian influenza viruses are characterized by significant genetic diversity. Deletions within the hemagglutinin (HA) receptor-binding domain represent a notable, albeit rare, evolutionary trajectory. Here, we systematically characterized the multifaceted impact of a single-amino-acid deletion at residue 227 of HA (designated HA_Δ227_), a naturally occurring deletion within the 220-loop that overlaps a key antigenic epitope, using isogenic reverse genetics H9N2 viruses. Paradoxically, the HA_Δ227_ imposed a fitness cost and was associated with enhanced HA receptor-binding avidity, reduced apparent virion-associated NA activity, altered HA/NA functional balance, attenuated viral replication *in vitro*, and reduced respiratory shedding *in vivo*. Notably, despite this compromised replicative fitness, infection with the mutant virus was associated with an altered host immune response in chickens, characterized by elevated levels of interferon-γ (IFN-γ), interleukin-10 (IL-10), interleukin-1β (IL-1β), and monocyte chemoattractant protein-1 (MCP-1). These altered immune responses were associated with more severe pulmonary lesions, indicating a dissociation between disease severity and viral RNA load. Furthermore, the deletion increased the pH threshold for HA-mediated membrane fusion and substantially altered antigenic relationships. Collectively, our findings identify HA residue 227 as a functionally important site at which a single-residue deletion is associated with a phenotypic trade-off between replicative capacity and disease severity: reduced replicative fitness accompanied by altered inflammatory responses and greater tissue injury. This study highlights the potential phenotypic significance of a rare deletion in influenza virus HA and demonstrates that viral burden alone may not adequately predict disease severity.

## Author summary

H9N2 avian influenza viruses present a persistent challenge to poultry health and a significant zoonotic threat. This study identifies a rare but potentially consequential evolutionary event—a single amino acid deletion at residue 227 of the viral hemagglutinin (HA) protein—and characterizes its complex and apparently contrasting biological effects. We demonstrate that this deletion imposes a cost on replicative fitness by disrupting the functional balance between HA and neuraminidase, thereby impairing viral replication. Paradoxically, the same deletion alters antigenic recognition and is associated with more severe disease and an inflammatory profile consistent with greater tissue injury. Our findings illustrate that a low-prevalence deletion in influenza virus HA may nevertheless be associated with notable phenotypic effects and that viral burden alone may not adequately predict disease severity. They also support complementing conventional measures of viral burden with immunopathological assessments when evaluating influenza variants with atypical phenotypes.

## Introduction

Influenza A viruses (IAVs) are RNA pathogens characterized by high mutation rates, posing a persistent and substantial threat to global public health. The IAV genome comprises eight segments of single-stranded, negative-sense RNA, encoding at least 22 proteins [1]. Hemagglutinin (HA), the major surface glycoprotein on the viral envelope, mediates the initial attachment of the virus to host cells by specifically recognizing and binding to sialic acid (SA) receptors. As the principal mediator of viral entry and the primary target of influenza vaccines, HA undergoes genetic and antigenic variation that can substantially influence viral fitness, transmissibility, and vaccine efficacy [2]. Neuraminidase (NA), another viral surface glycoprotein, catalyzes the cleavage of SA receptors from both host cells and newly formed virions. This enzymatic activity is crucial for the efficient release of progeny virus particles, preventing viral self-aggregation and receptor-mediated retention, thereby enabling the spread of infection. Given this indispensable role, NA is a well-established target for antiviral therapeutics [3].

The receptor-binding domain (RBD) constitutes the core functional region of the HA protein, where its specific amino acid sequence and three-dimensional conformation collectively determine viral recognition of and attachment to host cell receptors. Although SA receptors in avian, mammalian, and human hosts exhibit only subtle structural differences, the precise architecture of the RBD critically determines influenza virus receptor preference and binding avidity. This receptor-binding specificity represents a fundamental molecular determinant of cross‑species transmission and host adaptation, ultimately regulating the virus’s capacity to enter host cells and establish a productive infection [4–7]. Within the RBD, the highly conserved 220‑loop not only directs viral preference for human‑type α2,6‑linked SA receptors over avian‑type α2,3‑linked SA, but also forms a major antigenic epitope that is targeted by neutralizing antibodies across multiple HA subtypes [8].

H9N2 is among the most geographically widespread avian influenza virus (AIV) subtypes and circulates endemically in poultry across Asia and North Africa [8–10]. Beyond imposing substantial economic losses on the poultry industry, H9N2 poses a persistent public health concern owing to its proven capacity for cross‑species transmission [11,12]. Notably, despite decades of widespread vaccination in poultry, H9N2 viruses continue to circulate widely and remain predominant in chicken populations [10,13]. Persistent natural selection and host immune pressure have driven extensive genetic diversification in H9N2 viruses, primarily through the accumulation of amino acid substitutions and, to a lesser extent, through deletions in functionally critical regions [9,13–16]. These observations underscore the need to better understand the evolutionary and phenotypic consequences of mutations that may facilitate viral persistence. In this study, we investigated the multifaceted phenotypic impact of a naturally occurring single‑amino‑acid deletion at HA residue 227 (H3 numbering, hereafter designated HA_Δ227_). This residue is located within the RBD and overlaps a major antigenic epitope [5,8,12,17,18], positioning this deletion as a rare but potentially consequential evolutionary event. Using reverse genetics-derived isogenic viruses, we found that the HA_Δ227_ was associated with reduced replicative efficiency, altered antigenic recognition, and greater disease severity. This work provides insight into how specific viral deletion may be associated with a dissociation between viral replication and disease severity. These findings highlight the value of considering pathological and inflammatory outcomes alongside replicative fitness in influenza surveillance and risk assessment.

## Results

### Genetic and evolutionary characterization of an H9N2 virus harboring a deletion at HA residue 227

In June 2022, a commercial broiler chicken flock in Jilin Province, China, exhibited clinical signs including lethargy, anorexia, coughing, and audible respiratory rales. Mortality rose from 0.2% at 27–28 days of age to 1–1.6% by the time of market age. Postmortem examination revealed severe tracheal hemorrhages, excessive mucus secretion, pulmonary congestion, and fibrinous pericarditis, perihepatitis, airsacculitis, and peritonitis. RT-PCR and bacteriological culture of lung tissues confirmed a mixed infection involving AIV, infectious bronchitis virus, and *Escherichia coli*. Whole-genome sequencing identified the AIV as an H9N2 subtype, designated A/chicken/Jilin/WJ1/2022. Phylogenetic analysis showed that the HA gene of this isolate clustered within the B4 clade, which currently represents the predominant clade circulating in Chinese poultry [19], while its NA gene belonged to the contemporary prevailing N2.1 lineage [20] (Fig 1).

**Fig 1:**
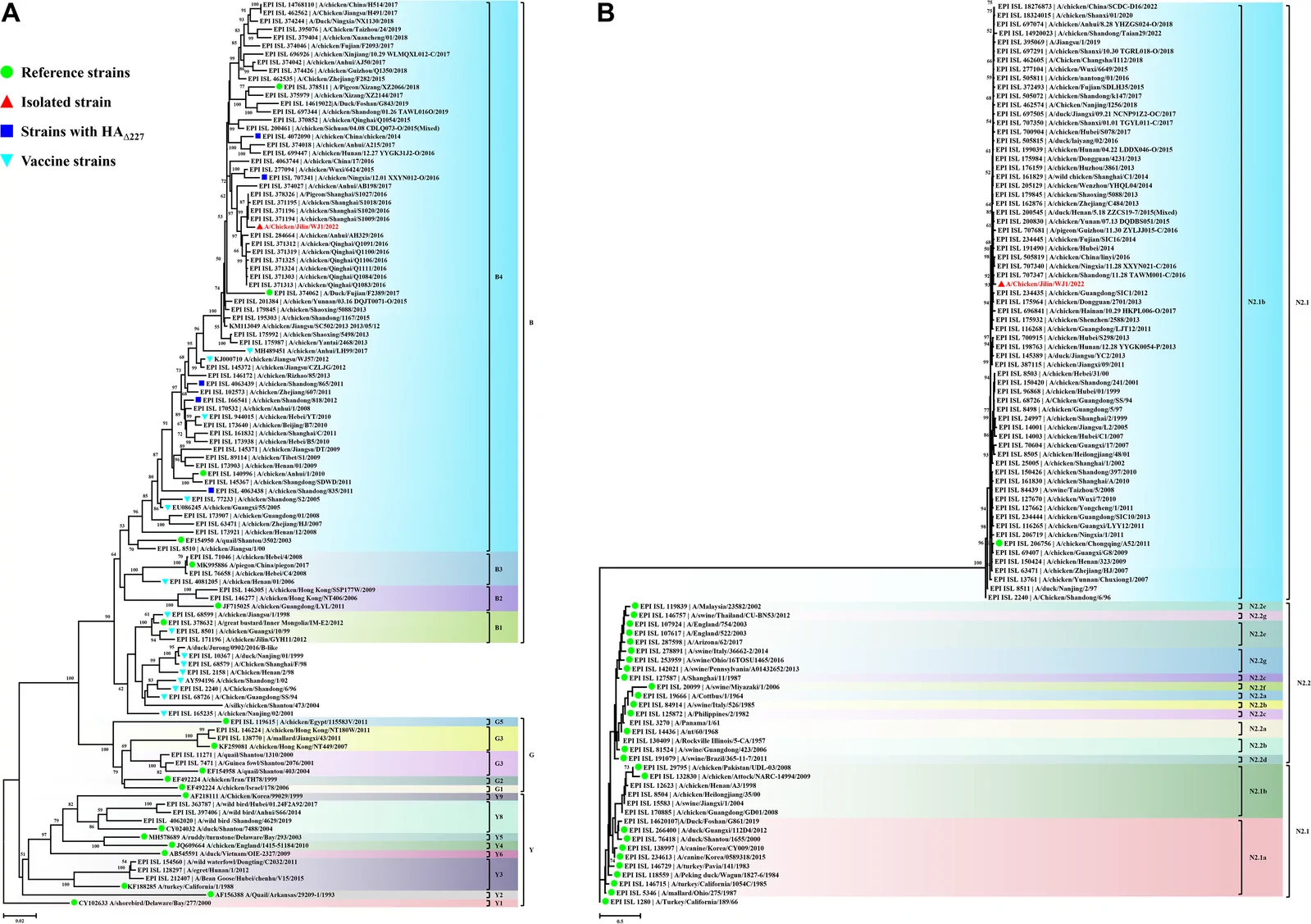
Phylogenetic analysis of HA and NA genes of H9N2 AIVs. Maximum-likelihood trees were constructed using HA (A) and NA (B) gene sequences of Chinese avian isolates in IQ-TREE v3.0.1 with 1,000 bootstrap replicates. For clarity, a subset of representative strains from each major clade was included to generate these simplified tree views. Key strains are annotated as follows: reference strains for each lineage (green circles), the isolate characterized in this study (red triangle with red text), other previously reported HA_Δ227_ variants (blue squares), and commercial H9N2 vaccine strains (light blue triangles). Bootstrap values ≥ 50% are displayed at corresponding nodes. Lineage classification and branch nomenclature adhere to the standards defined by Fusaro *et al*. [21] and Zhuang *et al*. [20].

### Conservation analysis of amino acid residues within the HA 220-loop of H9N2 viruses

The RBD of H9N2 hemagglutinin consists of four principal structural elements: the 130‑loop, 150‑loop, 190‑helix, and 220‑loop (Fig 2A–2C) [2]. Analysis of 7,056 publicly available HA sequences from H9N2 viruses isolated in China identified five additional viruses harboring the same deletion. Together with A/chicken/Jilin/WJ1/2022, these represented six known HA_Δ227_ viruses (Fig 2D). This finding indicates that the HA-227 deletion is extremely rare among H9N2 viruses. Conservation analysis of the 220‑loop region revealed that residue 227 is highly conserved, with methionine present in 85.7% and glutamine in 13.7% of the examined strains (Fig 2E). To evaluate evolutionary constraints, three codon-based selection analyses, including single-likelihood ancestor counting (SLAC), fast unconstrained Bayesian approximation (FUBAR), and fixed-effects likelihood (FEL), were applied to the 220-loop region. All three methods consistently indicated that site 227 is under negative selection (S1 Table), consistent with its high conservation and suggesting a critical functional role in HA. Structural superposition of the HA head domain of the HA_Δ227_ variant with that of the wild‑type HA yielded an overall C*α* root‑mean‑square deviation (RMSD) of 0.059 Å, indicating a highly conserved global fold. However, the deletion induced localized conformational shifts in five adjacent residues of the 220-loop compared to the Met227-containing HA: Leu222 (1.954 Å), Val223 (1.757 Å), Asn224 (3.894 Å), Gly225 (3.993 Å), and Leu226 (1.942 Å). Collectively, these displacements resulted in a subtle inward retraction of the 220‑loop in the HA_Δ227_ mutant (Fig 2F). Given the established role of the 220-loop in modulating receptor-binding avidity and antigenic properties [5,8,17,18], we hypothesized that deletion of residue 227 perturbs the local loop architecture, thereby influencing viral receptor engagement and immune‑escape potential. To experimentally assess the biological consequences of this deletion, we restored the conserved methionine at position 227 via site‑directed mutagenesis. Using reverse genetics, we then generated two isogenic recombinant viruses: reHA_Δ227_ (retaining the deletion) and reHA (with Met227 restored), enabling a direct comparison of phenotypic traits.

**Fig 2:**
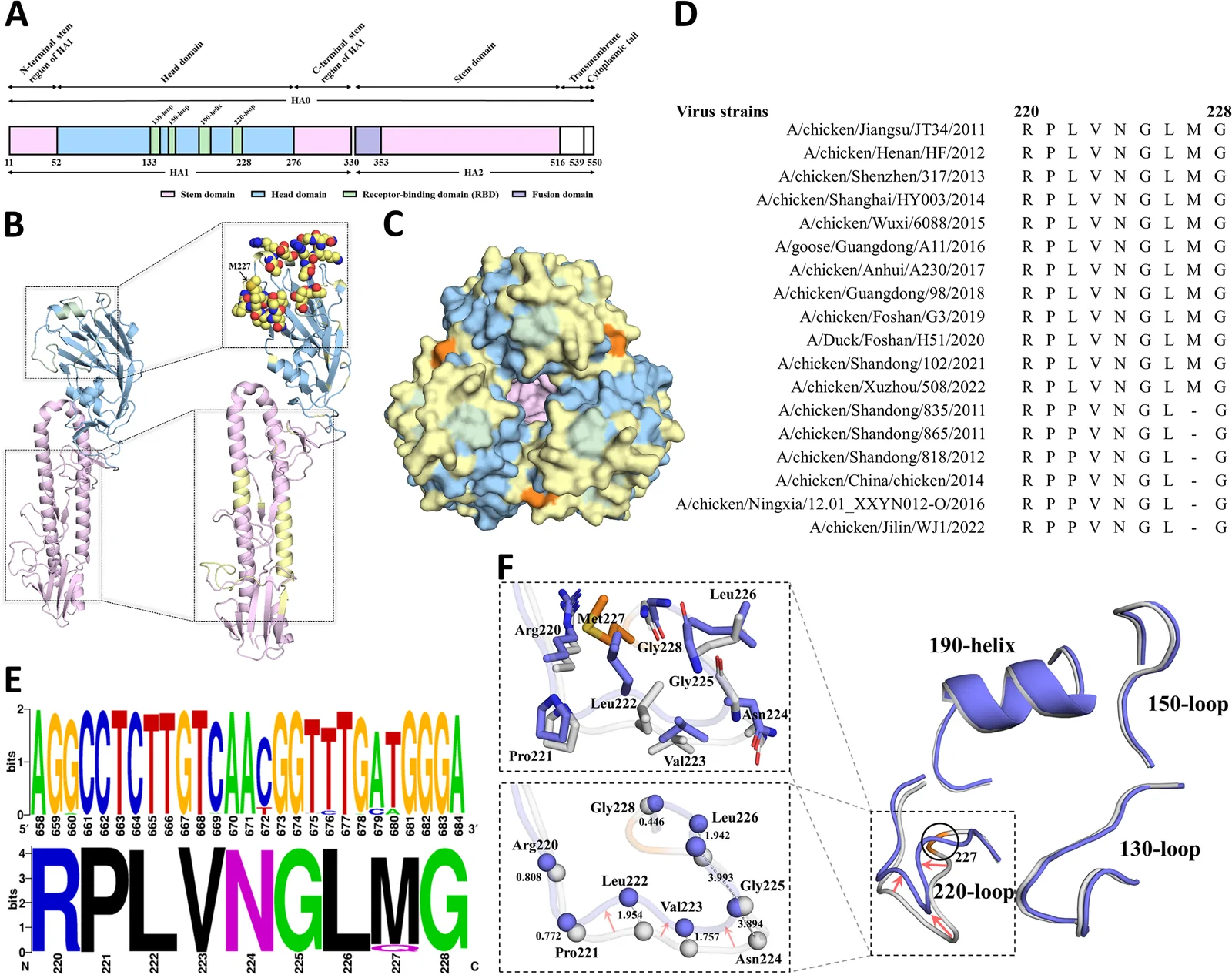
Structural features of the HA protein in H9 subtype influenza viruses. **(A)** Schematic representation of the H9 HA open reading frame, illustrating the four receptor-binding domain (RBD) elements. **(B)** Monomeric model of the HA ectodomain, with domains color-coded: head (blue), stem (pink), RBD (green), and antigenic sites (yellow). Antigenic sites within the RBD are shown as spheres, and residue 227 is highlighted with an arrow. **(C)** Spatial configuration of the four RBD elements within the HA trimer. RBD is shown in green, antigenic regions in yellow, and residue 227 in orange. **(D)** Multiple sequence alignment of the 220-loop region from six HA_Δ227_ isolates with available sequences. A dash (“-”) indicates the deletion of the residue at position 227. **(E)** Conservation analysis of the HA 220-loop amino acid sequence. **(F)** Structural superposition of HA_Δ227_ (blue) and its revertant counterpart (gray) with methionine (orange) restored at position 227 within the RBD. The deletion of residue 227 induces conformational rearrangement of the 220-loop, leading to spatial displacements in key residues: Leu222 (1.954 Å), Val223 (1.757 Å), Asn224 (3.894 Å), Gly225 (3.993 Å), and Leu226 (1.942 Å), as illustrated in stick representation. Gly225 and Gly228 are depicted as main-chain atoms only due to the absence of side chains.

### Deletion of HA residue 227 compromises acid stability and enhances the thermostability of H9N2 AIV

The efficient transmission and epidemic spread of IAVs rely on their ability to withstand environmental stressors, including acidic conditions and temperature fluctuations. Viral stability under low pH (acid stability) and at elevated temperatures (thermostability) critically affects viral entry, replication, and overall infectivity, thereby making these traits important determinants of viral fitness and epidemic potential [22,23]. To assess the effect of the HA_Δ227_ on acid stability, viruses were incubated in buffers with different pH values, and hemagglutination activity was subsequently measured. Both recombinant viruses showed a progressive reduction in hemagglutination titers as pH decreased (Fig 3A). At pH 6.0, reHA_Δ227_ exhibited significantly lower titers than reHA. By pH 5.0, reHA_Δ227_ had completely lost hemagglutination activity, whereas reHA still retained detectable activity, although reduced by fourfold, indicating that acid stability was severely compromised in the deletion mutant. In contrast, reHA_Δ227_ showed marked thermostability. After 24 h at 37 °C or 42 °C, reHA lost detectable hemagglutination activity, while reHA_Δ227_ maintained detectable hemagglutination activity for up to 120 h at 37 °C and 48 h at 42 °C (Fig 3B–3C).

**Fig 3:**
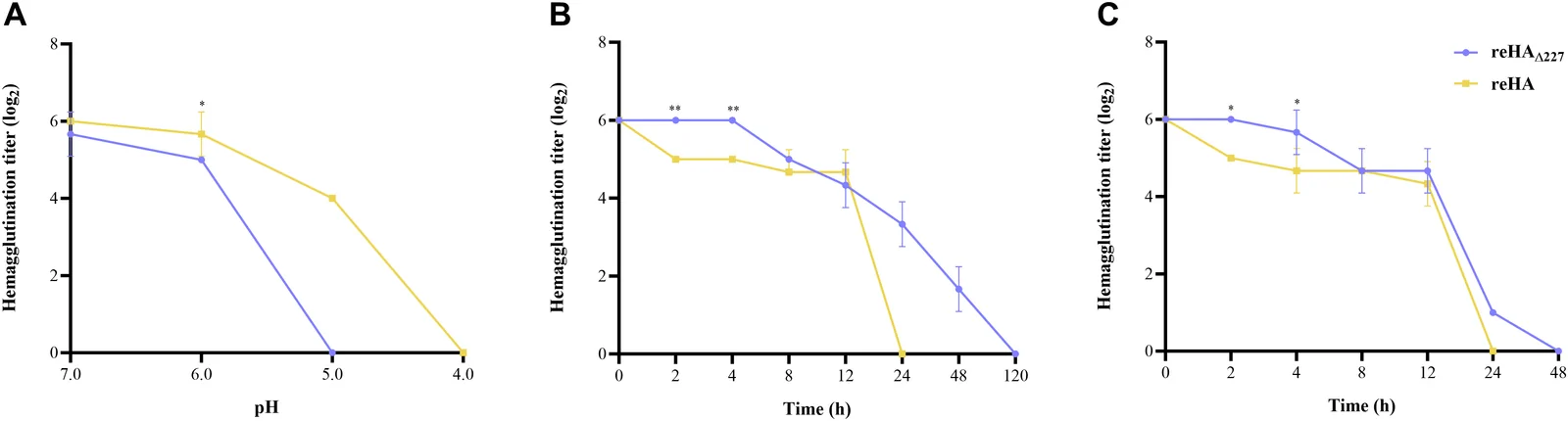
Acid and thermal stability of H9N2 viruses. **(A)** Acid stability was determined by incubating virus samples (initial hemagglutination titer = 6 log_2_) in buffers adjusted to the indicated pH values at 37 °C for 10 min, followed by measurement of residual hemagglutination activity. **(B, C)** Thermostability was assessed by incubating virus aliquots (initial hemagglutination titer = 6 log_2_) at 37 °C (B) or 42 °C (C) for the indicated durations, with hemagglutination titers measured at each time point to derive inactivation kinetics. Data are shown as the mean ± SD from three independent experiments.

### Deletion of HA residue 227 increases the pH threshold for HA-mediated membrane fusion of H9N2 AIV

IAVs enter host cells primarily through SA-mediated endocytosis. Following internalization, fusion of the viral envelope with the endosomal membrane is mediated by HA, which is initially maintained in a metastable prefusion conformation. Acidification below a critical pH within the endosome triggers irreversible conformational rearrangement in HA, culminating in membrane fusion [24]. However, premature exposure to acidic conditions can irreversibly inactivate HA and thereby reduce viral infectivity [25,26]. The acid stability of HA varies among influenza strains and is inversely correlated with the pH threshold required for fusion; in general, a lower fusion pH reflects greater acid stability [24]. To determine whether the HA_Δ227_ altered the pH threshold for HA-mediated membrane fusion, infected Vero cells were exposed to buffers ranging from pH 5.0 to 6.1 at 0.1-unit intervals, and syncytium formation was assessed. The highest pH supporting reproducible syncytium formation was 5.7 for reHA_Δ227_ and 5.3 for reHA in all three independent experiments (Fig 4). Thus, the HA_Δ227_ increased the fusion pH threshold by 0.4 pH units.

**Fig 4:**
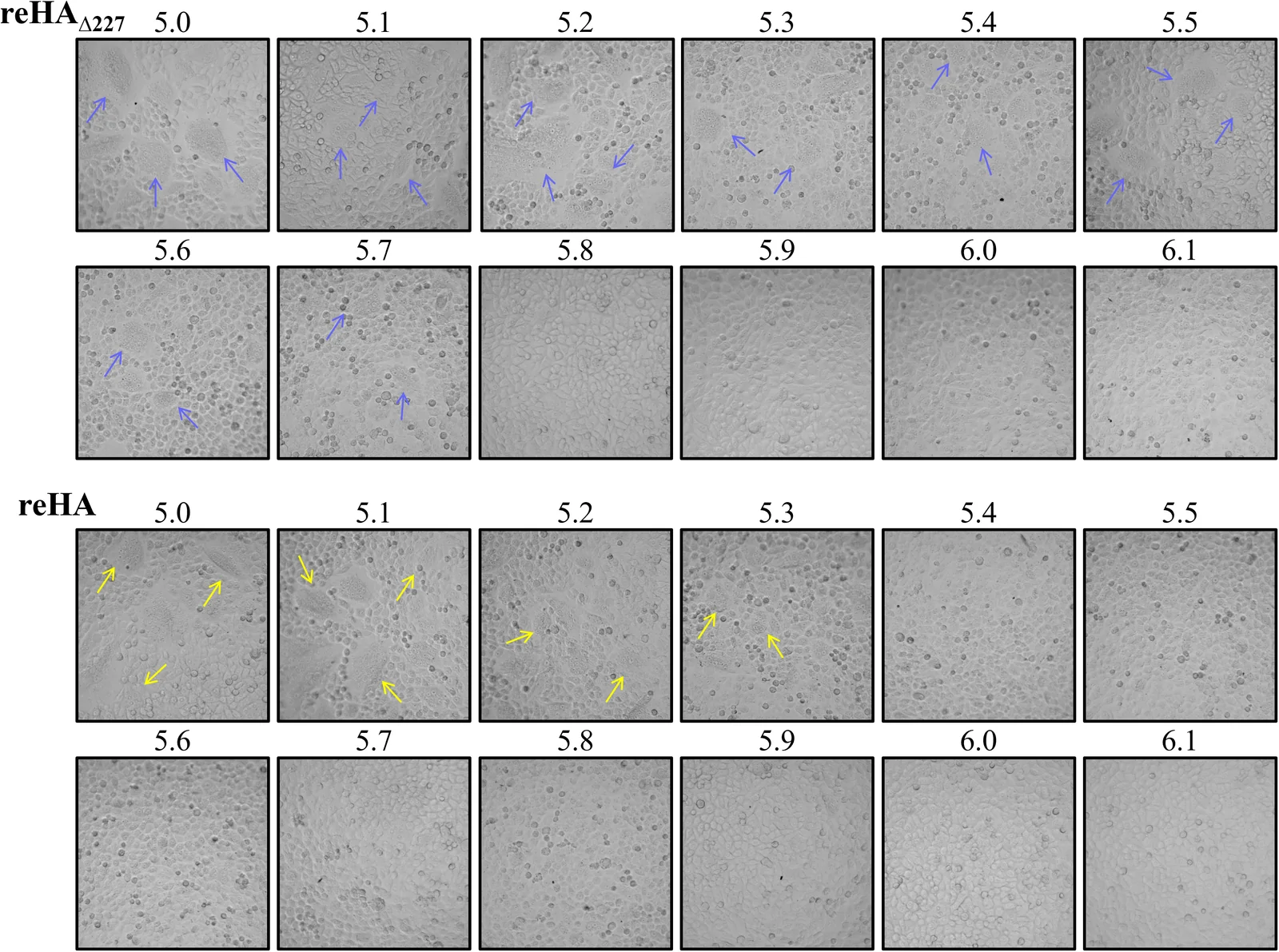
Determination of the pH threshold for H9N2 virus HA-mediated membrane fusion. Vero cells were infected with the indicated viruses at a multiplicity of infection (MOI) of 3 for 16–20 **h.** After incubation, the medium was replaced with serum‑free DMEM containing 5 μg/mL TPCK-treated trypsin for 15 min. The cells were then exposed to prewarmed fusion buffers at the indicated pH values for 15 min, returned to complete medium for 3 h, and fixed with 4% paraformaldehyde. Syncytium formation was evaluated in five randomly selected fields per condition, with a syncytium defined as a fused cell containing at least five nuclei. The fusion pH threshold was defined as the highest pH at which reproducible syncytium formation was observed across three independent experiments. The same threshold was obtained for each virus in all three independent experiments. Arrows indicate representative syncytia. Images are representative of one of three experiments. Magnification, × 400.

### Deletion of HA residue 227 enhances receptor-binding avidity of H9N2 AIV

The RBD of HA plays a pivotal role in initiating the IAV life cycle [5,8]. To evaluate the effect of the HA_Δ227_ on receptor‑binding properties, we compared the avidity of reHA_Δ227_ and reHA for two sialylated glycan analogs using a solid‑phase binding assay. The well-characterized H9N2 virus A/Quail/Hong Kong/G1/97 (G1) was included as a reference strain. The results showed that both reHA_Δ227_ and reHA exhibited stronger binding to the human‑type receptor analog Neu5Acα2,6Galβ1,4GlcNAc (6′SLN) than to the avian‑type Neu5Acα2,3Galβ1,4GlcNAc (3′SLN). Compared with reHA, reHA_Δ227_ showed lower apparent *K*_D_ values for both receptor analogs, indicating greater apparent receptor-binding avidity (Fig 5A–5B and S2 Table). To relate these functional differences to potential structural alterations, molecular docking simulations were performed. For 6′SLN, interactions involved residues Thr135, Arg156, and Gly228 in both HA variants, although the ligand adopted a slightly altered binding pose in the HA_Δ227_ model (Fig 5C). Binding to 3′SLN involved Asp133, Thr135, and Arg156, with largely conserved binding conformations between the two variants (Fig 5D). The calculated binding energies were similar: −7.0 kcal/mol for HA_Δ227_ and −6.9 kcal/mol for HA with 6′SLN, and −6.5 kcal/mol for both variants with 3′SLN. Hemagglutination assays further supported greater receptor avidity for reHA_Δ227_. After virus suspensions standardized to 256 hemagglutination units (HAU) were incubated with 0.5% chicken red blood cells (RBCs) on ice for 1 h, reHA_Δ227_ showed a one-log_2_ reduction in hemagglutination activity, whereas reHA showed a two-log_2_ reduction (Fig 5E). To quantify viral attachment independently of NA activity, we first determined the half-maximal inhibitory concentration (IC_50_) of the NA inhibitor zanamivir. The IC_50_ values were 2.00 nM for reHA_Δ227_ and 2.16 nM for reHA (S1 Fig), indicating comparable susceptibility to zanamivir between them. A549 and MDCK cells were then exposed to virus at multiplicities of infection (MOIs) of 10 and 0.1, respectively, in the presence of zanamivir at 10 × IC_50_ to completely suppress NA activity. Viral attachment was assessed after incubation at 4 °C for 2 h by immunofluorescence and quantification of cell-associated infectious virus. reHA_Δ227_ produced significantly greater cell-associated viral signals and titers than reHA (Fig 5F–5H), consistent with its greater apparent receptor-binding avidity.

**Fig 5:**
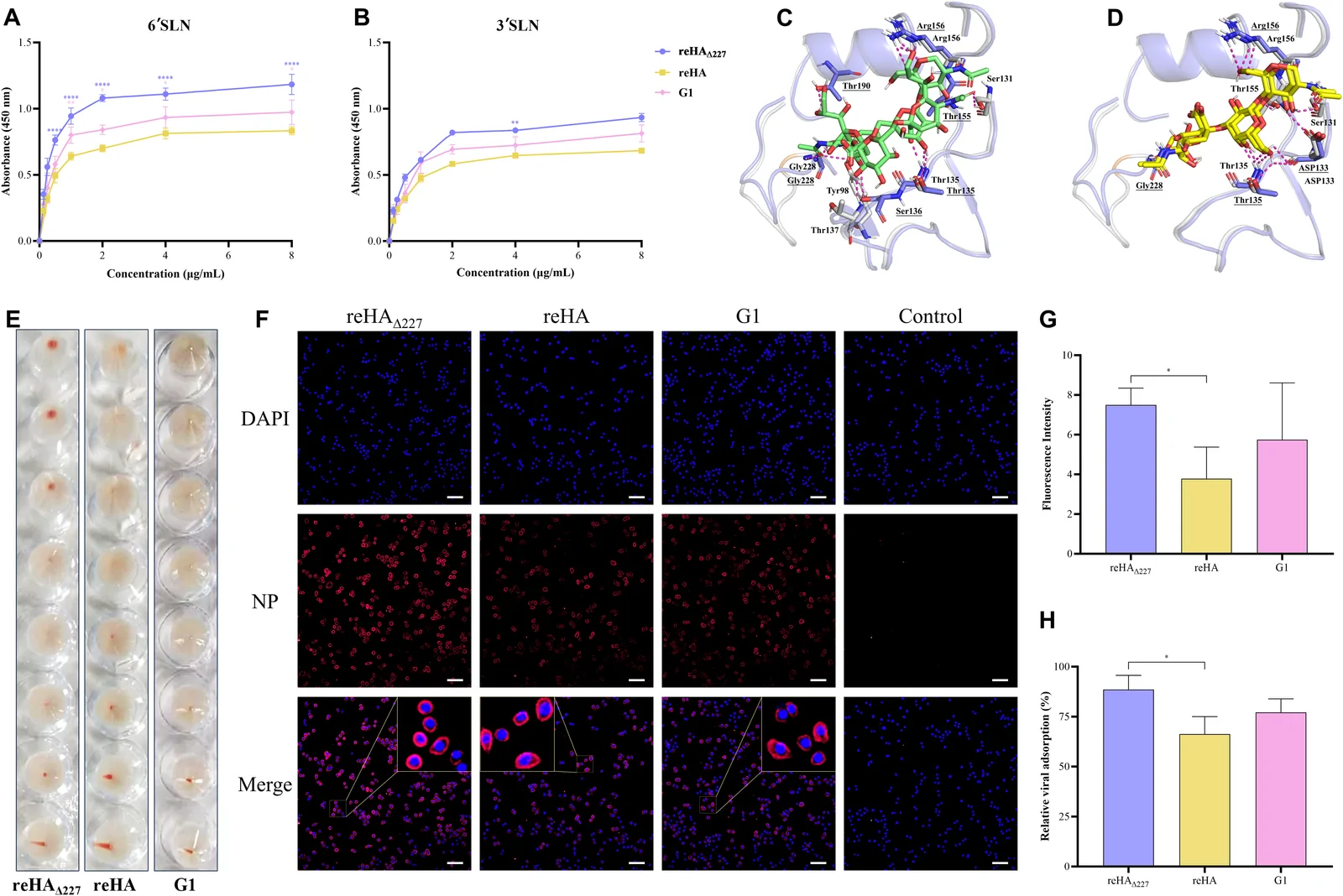
HA receptor-binding avidity and interactions of H9N2 virus HA. **(A, B)** Solid-phase receptor-binding avidity of the indicated viruses for the human-type receptor analog 6′SLN (A) and the avian-type receptor analog 3′SLN **(B)**. Asterisks indicate statistically significant differences relative to reHA. **(C, D)** Molecular docking and structural superposition of HA_Δ227_ (light blue) and HA (gray) in complex with 6′SLN (ID: PRD900046, C) or 3′SLN (ID: PRD900067, **D)**. The receptor-interacting residues are shown in stick representation; hydrogen bonds are indicated in magenta, and relevant HA residues are labeled. Underlined residues denote interactions unique to HA_Δ227_. **(E)** Representative results of the hemagglutination assay. Viruses with an initial titer of 256 hemagglutination units were serially twofold diluted and incubated with 0.5% chicken erythrocytes on ice for 1 h, after which endpoint hemagglutination titers were recorded. **(F–H)** Virus adsorption assay. Viruses pretreated with zanamivir at 10 × IC_50_ were incubated with A549 cells at a multiplicity of infection (MOI) of 10 or MDCK cells at an MOI of 0.1 for 2 h at 4 °C in the absence of TPCK-treated trypsin. Virions bound to the A549 cell surface were detected via immunofluorescence staining of viral nucleoprotein (NP, red), with nuclei counterstained in blue **(F)**, and fluorescence intensity was quantified using ImageJ **(G)**. Scale bar, 100 μm. In parallel, infectious virions bound to the MDCK cell surface were quantified by the 50% tissue culture infectious dose (TCID_50_) **(H)**. Data are shown as the mean ± SD from three independent experiments.

### Deletion of HA residue 227 alters virion-associated NA activity and receptor-release kinetics

A functional balance between HA-mediated receptor binding and NA-mediated receptor cleavage is critical for IAV fitness; alterations in HA receptor avidity are often counterbalanced by adaptive changes in NA enzymatic activity [9,27]. To evaluate the impact of the HA_Δ227_ on NA function, we first performed an erythrocyte elution assay. At 33 °C, reHA initially displayed higher hemagglutination titers than reHA_Δ227_ during the first 210 min, but its activity subsequently declined below that of reHA_Δ227_ and was completely lost after 240 min of incubation (Fig 6A). At 37 °C and 42 °C, the hemagglutination activity of reHA remained consistently lower than that of reHA_Δ227_ and was entirely abolished within 30 min (Fig 6B and 6C), indicating that higher temperatures substantially accelerate NA‑dependent erythrocyte elution. In contrast, reHA_Δ227_ retained detectable hemagglutination activity for up to 360 min at all temperatures tested. These erythrocyte-elution profiles indicated that reHA_Δ227_ exhibited the slowest receptor-release phenotype, consistent with a shift in the functional balance between HA-mediated attachment and NA-mediated receptor cleavage. To further characterize NA function, virion preparations were adjusted to contain equivalent amounts of NA protein based on western blot densitometry (S2 Fig), after which NA enzymatic activity was assessed using monovalent and multivalent substrates. MUNANA-based kinetic analysis showed that, after normalization to the estimated NA protein abundance, reHA_Δ227_ virion preparations exhibited a higher apparent *K*m and lower apparent *V*max/*K*m than reHA (Fig 6E, S3 Table). Correspondingly, in an enzyme-linked lectin assay (ELLA), reHA_Δ227_ removed α2,3-linked SAs from fetuin less efficiently than reHA, as detected using MAL I (Fig 6F). Collectively, these results showed that reHA exhibited higher NA activity than reHA_Δ227_.

**Fig 6:**
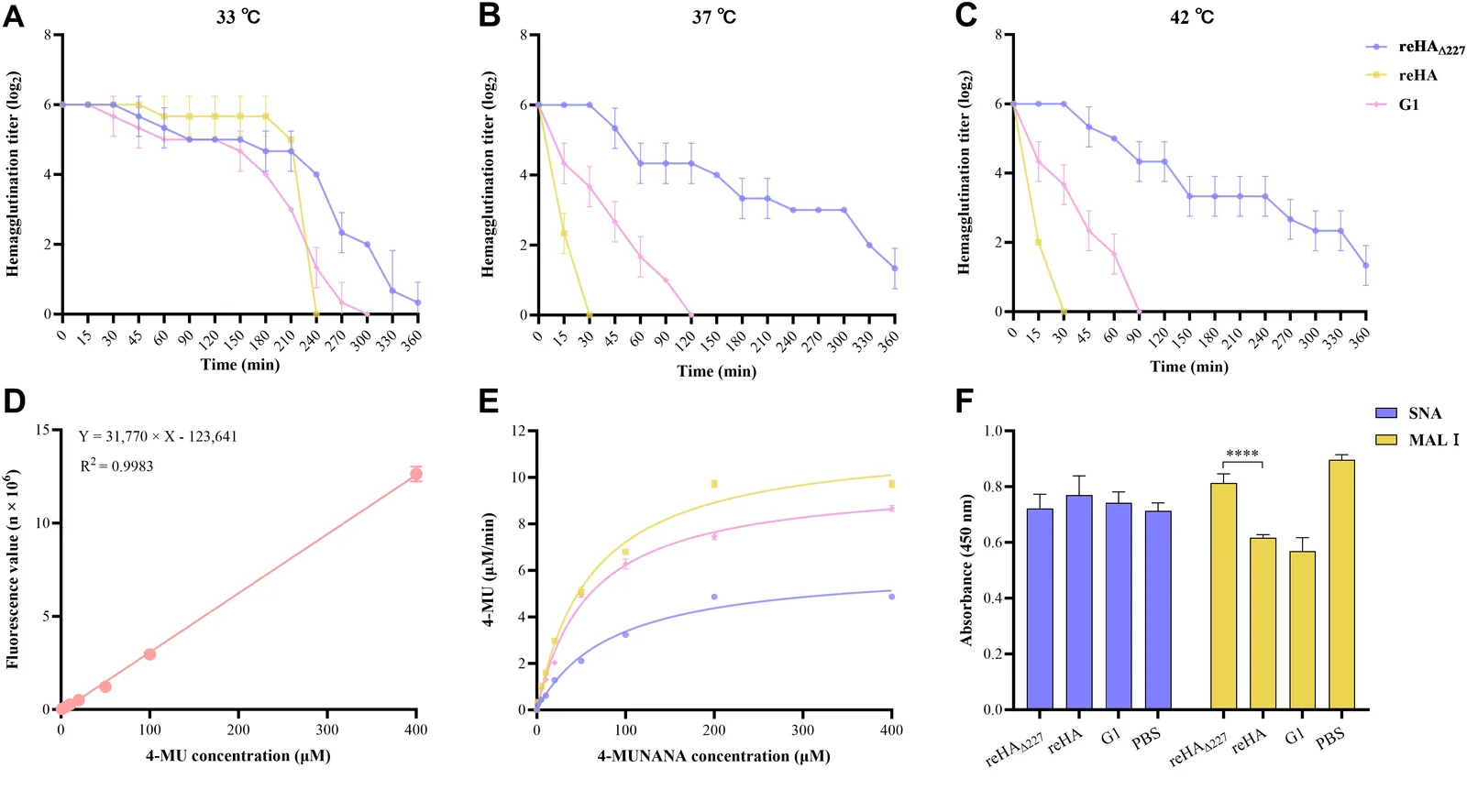
NA enzymatic activity of H9N2 viruses. **(A–C)** NA‑dependent erythrocyte elution kinetics. Serially diluted viruses were mixed with 0.5% chicken erythrocytes and adsorbed at 4 °C for 1 **h.** Elution was monitored at 33 °C **(A)**, 37 °C **(B)**, or 42 °C (C) over 6 h by measuring residual hemagglutination titers at indicated time points to evaluate temperature‑sensitive NA activity. **(D)** Standard curve of 4-methylumbelliferone (4-MU). Serial dilutions of 4-MU were prepared in a black 96-well plate, and fluorescence intensity was measured (excitation: 365 nm; emission: 450 nm). A linear regression model was fitted using GraphPad Prism v10.1 to quantify the amount of released 4-MU. **(E)** Kinetic analysis of NA activity using the monovalent substrate 2′-(4-methylumbelliferyl)-α-D-N-acetylneuraminic acid (4-MUNANA). Viruses containing equivalent amounts of NA protein were incubated with serially diluted 4-MUNANA, and the fluorescence of released 4-MU was recorded. Michaelis–Menten kinetic parameters, including the maximum reaction velocity (*V*_max_) and Michaelis constant **(*K***_**m**_), were derived through nonlinear regression in GraphPad Prism v10.1. **(F)** NA activity toward the multivalent substrate fetuin. Fetuin-coated plates were washed, incubated with viruses containing equivalent amounts of NA protein at 37 °C for 18 h, and then probed with biotinylated Sambucus nigra lectin (SNA, specific for α2,6-linked sialic acid) or Maackia amurensis lectin I (MAL I, specific for α2,3-linked sialic acid) for 2 h at 37 °C. After incubation with HRP-conjugated streptavidin and TMB substrate, the reaction was stopped with H_2_SO_4_, and absorbance was measured at 450 nm. Data are shown as the mean ± SD from three independent experiments.

### Deletion of HA residue 227 impairs viral replication in mammalian and avian cell models

To evaluate the effect of the HA_Δ227_ on viral replication, one-step and multistep growth curves were generated in MDCK and CEF cells. In MDCK cells, one-step growth analysis showed that reHA was first detected at 6 hours post-infection (hpi), whereas reHA_Δ227_ remained undetectable until 8 hpi, suggesting delayed early replication. Thereafter, both viruses replicated rapidly. The reHA titer increased by approximately 3.8 log_10_ 50% tissue culture infectious dose (TCID_50_)/ 0.1 mL from 4 to 8 hpi, whereas the reHA_Δ227_ titer increased by approximately 3.6 log_10_ TCID_50_/0.1 mL from 6 to 8 hpi and peaked at 10 hpi (Fig 7A). A similar pattern was observed in the multistep growth analysis. reHA replicated more rapidly, reaching approximately 7.0, 10.0, and 11.2 log_10_ TCID_50_/0.1 mL at 12, 24, and 36 hpi, respectively. In contrast, reHA_Δ227_ reached only 3.9 log_10_ TCID_50_/0.1 mL at 12 hpi and subsequently increased to approximately 8.7 and 9.6 log_10_ TCID_50_/0.1 mL at 24 and 36 hpi, respectively, before gradually declining. Although the difference between the two viruses narrowed at later time points, reHA exhibited significantly higher titers than reHA_Δ227_ at 12 and 36 hpi (Fig 7C). Collectively, these results indicate that the HA_Δ227_ primarily delayed viral replication during the early phase of infection in MDCK cells. In CEF cells, one-step growth analysis showed that both viruses had titers of approximately 6.0 log_10_ TCID_50_/0.1 mL at 2 hpi, which remained relatively stable through 4 hpi. From 4 to 6 hpi, the reHA titer increased more rapidly than that of reHA_Δ227_ and subsequently declined. By contrast, the reHA_Δ227_ titer continued to increase modestly from 6 to 8 hpi, by approximately 0.4 log_10_ TCID_50_/0.1 mL, and approached that of reHA at 8–10 hpi. Nevertheless, reHA exhibited significantly higher titers than reHA_Δ227_ at both 6 and 8 hpi (Fig 7E). In the multistep growth assay, reHA replicated more rapidly during the first 24 h, with titer increases of 4.4 and 3.1 log_10_ TCID_50_/0.1 mL during 0–12 and 12–24 hpi, respectively. Although reHA_Δ227_ exhibited a greater increase than reHA from 24 to 36 hpi, its titer subsequently declined. In contrast, the reHA titer continued to increase, peaked at 48 hpi, and then declined. Except at 36 hpi, reHA exhibited significantly higher titers than reHA_Δ227_ at all measured post-infection time points (Fig 7G). These results further indicate that the HA_Δ227_ impaired viral replication in CEF cells, particularly during the early phase. Overall, the HA_Δ227_ delayed and reduced early viral replication in both MDCK and CEF cells. Although the replication of reHA_Δ227_ partially recovered at later time points, it produced lower overall virus yields than reHA, as indicated by its lower peak titers and smaller areas under the curves (AUCs) (Fig 7B, 7D, 7F, and 7H).

**Fig 7:**
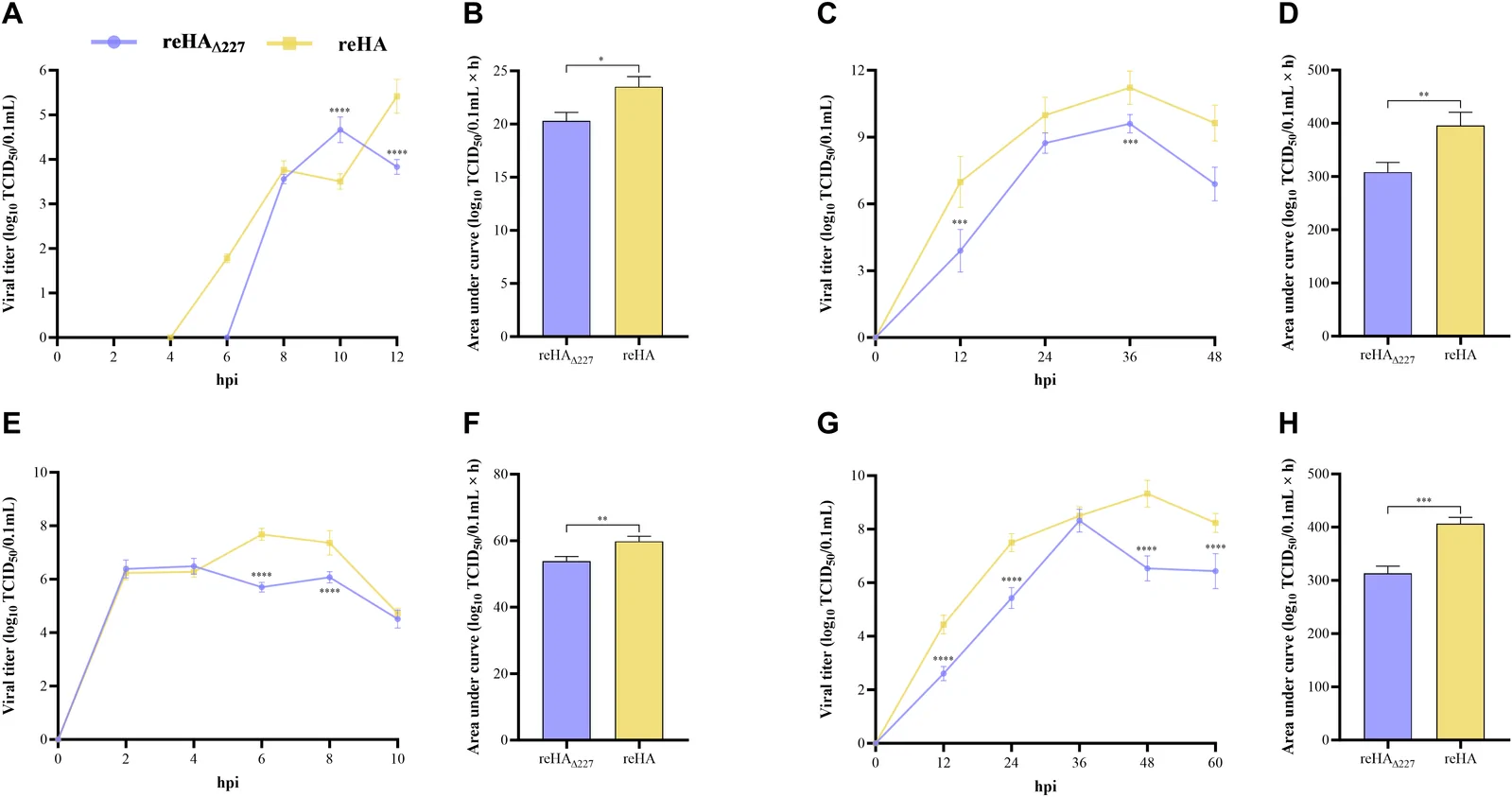
*In vitro* replication kinetics of H9N2 viruses. Viral replication was evaluated in MDCK and chicken embryo fibroblast (CEF) cells using one-step (MOI = 0.1) and multistep (MOI = 0.01) growth curves. One-step growth curves and the corresponding areas under the curve (AUCs) are shown for MDCK cells in (A, B) and CEF cells in **(E, F)**, respectively; multistep growth curves and the corresponding AUCs are shown for MDCK cells in (C, D) and CEF cells in **(G, H)**, respectively. Viral titers are expressed as log_10_ TCID_50_/0.1 mL. Data are presented as the mean ± SD of three independent experiments. hpi, hours post-infection.

### Deletion of HA residue 227 alters antigenic reactivity of H9N2 AIV

Residue 227 in HA plays a dual role in modulating both receptor-binding avidity and antigenicity of IAVs [5,8,12,17,18]. To evaluate the antigenic impact of the HA_Δ227_, we performed cross-hemagglutination inhibition (HI) assays using chicken polyclonal antisera raised against reHA_Δ227_ and reHA. Both antisera displayed higher HI titers against the homologous virus than against the heterologous strain. Specifically, reHA_Δ227_ antiserum exhibited a 6–7 log_2_ reduction in HI titer when tested against reHA, while reHA antiserum showed a milder reduction (1–3 log_2_) against reHA_Δ227_ (S4 Table). After standardizing HI titers (mean = 0, SD = 1), we calculated the antigenic distance between the two viruses, which was 4.32 units.

### Deletion of HA residue 227 is associated with greater disease severity despite a lower viral burden

To define the contribution of HA residue 227 to pathogenicity and transmission in chickens, 3-week-old SPF chickens were assigned to the reHA_Δ227_, reHA, and mock groups. For each virus, 12 chickens were inoculated intranasally with 10^6^ TCID_50_ of virus in 100 μL of PBS, and six naïve chickens were included as direct-contact birds; 12 additional chickens received PBS as mock controls. At 1, 3, 5, and 7 days post-inoculation (dpi), three inoculated chickens from each virus group and three mock-inoculated chickens were euthanized for terminal sampling. The group allocation, sampling schedule, and numbers of chickens included in each analysis are detailed in the Methods. Across the sequentially sampled inoculated cohorts (n = 3 per virus at each time point), mean oropharyngeal viral RNA loads increased from 1 to 5 dpi and declined at 7 dpi in both virus groups. At each time point, however, reHA_Δ227_-inoculated chickens had lower mean viral RNA loads than reHA-inoculated chickens, with significant between-virus differences at 1, 3, 5, and 7 dpi (Fig 8A). Viral RNA was also detected longitudinally in direct-contact birds (n = 6 per virus group), demonstrating transmission under the co-housing conditions, although the magnitude and kinetics of shedding differed between the two viruses. Cloacal shedding was lower and less consistent than oropharyngeal shedding; nevertheless, reHA_Δ227_-inoculated chickens again exhibited lower viral RNA loads than reHA-inoculated chickens at 5 and 7 dpi (Fig 8B). This lower viral RNA burden extended to the sampled organs. In sequentially euthanized chickens (n = 3 per virus at each time point), mean viral RNA loads in the lung and duodenum peaked at 5 dpi and declined at 7 dpi, but remained significantly lower in reHA_Δ227_-infected chickens than in reHA-infected chickens at 3, 5, and 7 dpi (Fig 8C, 8D). Thus, the HA_Δ227_ was associated with a lower viral RNA burden in both respiratory and intestinal tissues. Despite this lower viral RNA burden, reHA_Δ227_ infection was not associated with attenuated disease. Although both viruses caused respiratory signs, including sneezing and nasal discharge, reHA_Δ227_ was associated with higher clinical scores. Among inoculated chickens prespecified for longitudinal clinical monitoring through 7 dpi (n = 3 per virus), scores began to diverge between the two virus groups at 1 dpi, peaked at 5 dpi, and remained higher in the reHA_Δ227_ group through 7 dpi (Fig 8E). Moreover, among the chickens prespecified for longitudinal body-weight monitoring, both inoculated and direct-contact chickens in the reHA_Δ227_ group gained less weight than their respective reHA counterparts (n = 3 chickens per category per virus group), whereas mock-inoculated chickens (n = 3) showed sustained weight gain throughout the observation period (Fig 8F). Consistent with these clinical findings, blinded histopathological assessment at 5 dpi (n = 3 chickens per group) revealed more severe pulmonary injury in reHA_Δ227_-infected chickens than in both reHA-infected and mock-inoculated chickens (Fig 8G). Duodenal lesion scores showed the same directional difference, with reHA_Δ227_ producing more severe mucosal injury than reHA (Fig 8H). Pulmonary and duodenal histopathological changes are shown in the supplementary figures available at https://figshare.com/s/879f4dfa64cdab3b4521, with the corresponding field-level scores and morphological rationales provided in S5 and S6 Tables. The respective immunofluorescence findings are presented in S3 and S4 Figs. Collectively, these findings reveal a phenotypic dissociation in which reHA_Δ227_ caused greater clinical and tissue injury despite producing a lower viral RNA burden. We next examined whether this discordant phenotype was accompanied by altered inflammatory responses. In sequentially sampled chickens (n = 3 per virus at each time point), serum IFN-γ and IL-10 concentrations were higher following reHA_Δ227_ infection than following reHA infection from 3 to 7 dpi (Fig 9A, 9B). This difference was more pronounced in lung homogenates, in which reHA_Δ227_ elicited higher IFN-γ and IL-10 concentrations over the same interval (Fig 9C, 9D). Pulmonary IL-1β and MCP-1 concentrations also increased sharply after 1 dpi, peaked at 5 dpi, and remained elevated at 7 dpi, with consistently higher levels in the reHA_Δ227_ group (Fig 9E, 9F).

**Fig 8:**
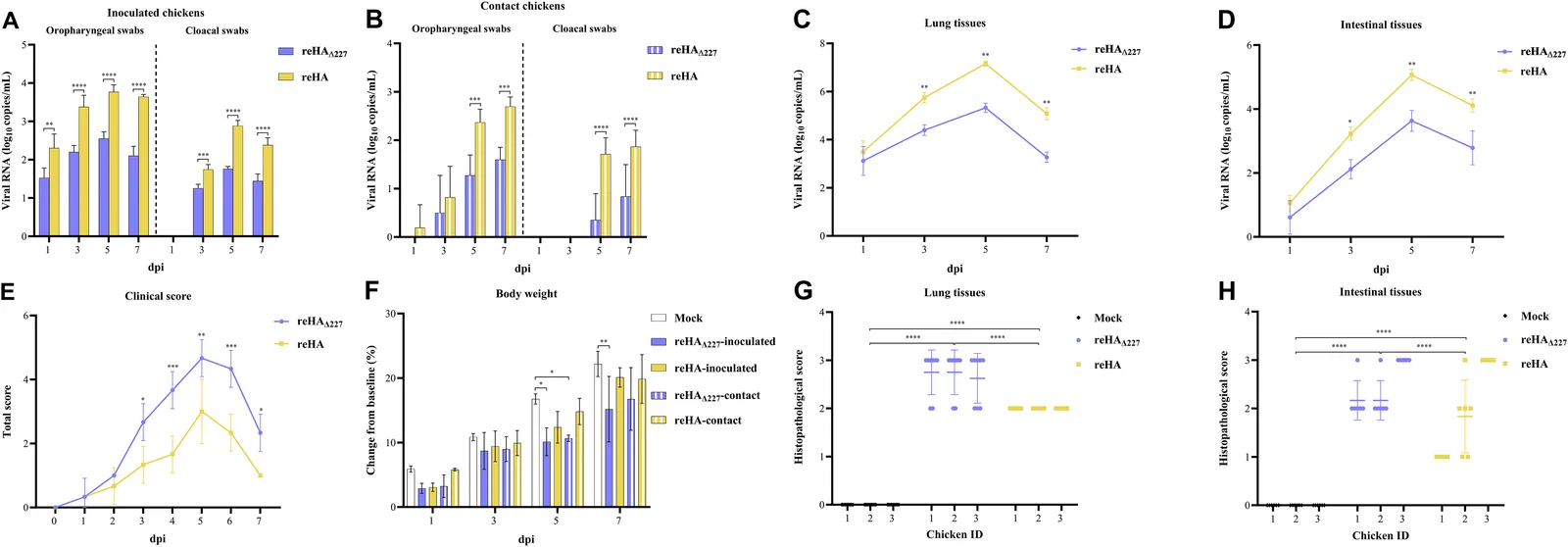
Viral loads, clinical manifestations, body-weight changes, and histopathological lesions in H9N2 virus-infected chickens. Three-week-old SPF chickens were assigned to the reHA_Δ227_, reHA, or mock group. Each virus group (n = 18) comprised 12 chickens intranasally inoculated with 10^6^ TCID_50_ of the corresponding virus and six naïve chickens housed in direct contact with the inoculated birds. Chickens in the mock group (n = 12) received an equivalent volume of PBS. Three inoculated chickens from each virus group and three mock chickens were euthanized at 1, 3, 5, and 7 dpi for terminal sample collection. **(A, B)** Viral RNA loads in oropharyngeal (A) and cloacal (B) swabs collected from inoculated chickens immediately before euthanasia (n = 3 per group per time point) and longitudinally from direct-contact chickens (n = 6 per group), as determined by RT–qPCR. **(C, D)** Viral RNA loads in lung (C) and duodenal (D) tissues collected from inoculated chickens (n = 3 per group per time point). **(E)** Longitudinal clinical scores of inoculated chickens assigned *a priori* to the 7-dpi terminal sampling cohort (n = 3 per group), assessed across six categories: demeanor and activity, feather condition and posture, respiratory signs, nasal and ocular signs, feed and water intake, and fecal appearance. **(F)** Changes in body weight relative to the day 0 baseline in longitudinally monitored inoculated and direct-contact chickens (n = 3 per virus and exposure group). Individual chickens were prespecified for longitudinal monitoring and followed throughout the observation period. **(G, H)** Histopathological scores of H&E-stained lung (G) and duodenal (H) sections collected from inoculated chickens at 5 dpi (n = 3 per group). Histopathological evaluation was performed in a blinded manner as described in the Methods. Data are presented as the mean ± SD. dpi, days post-inoculation; ID, identification.

**Fig 9:**
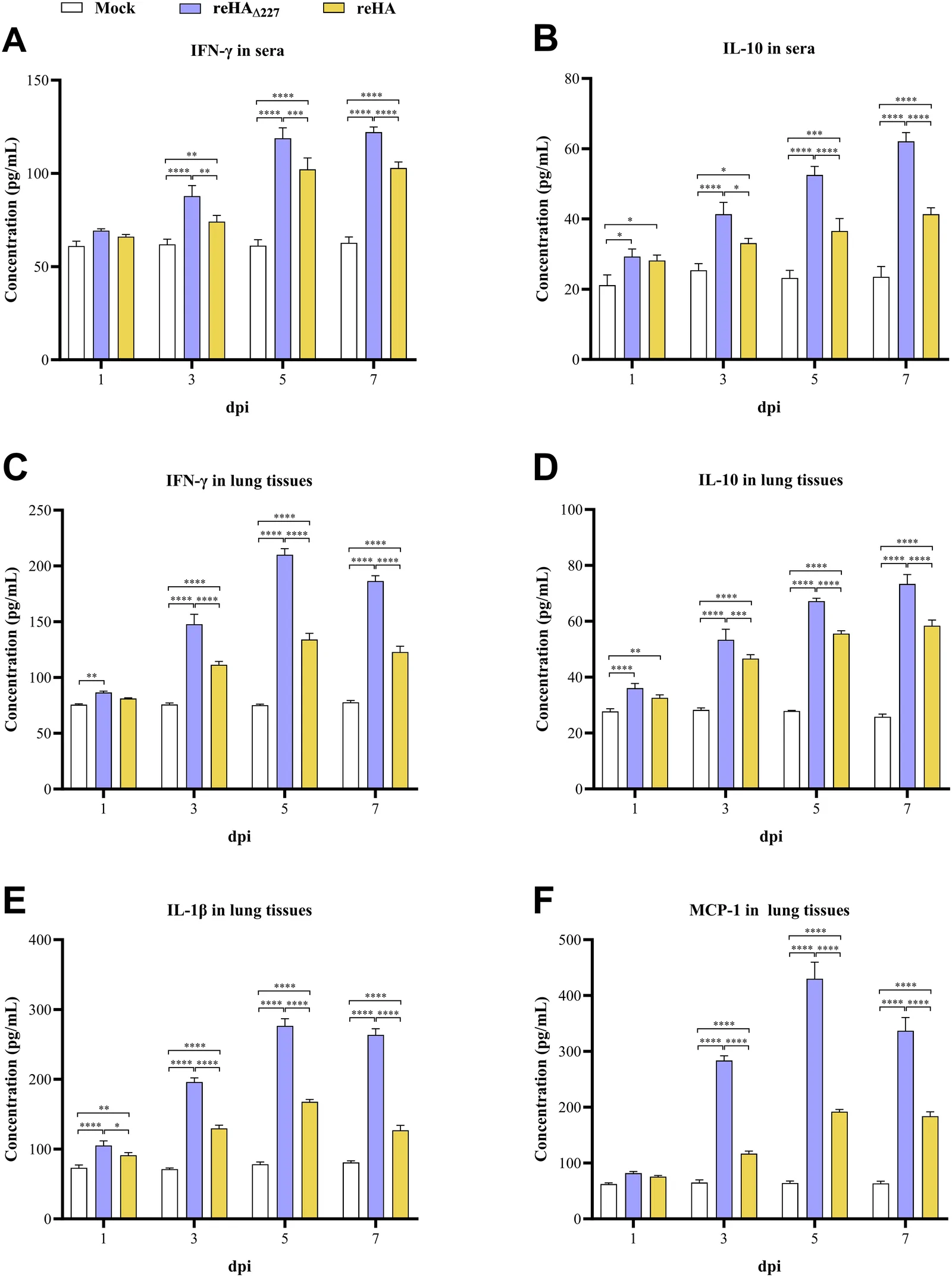
Cytokine and chemokine responses following H9N2 virus infection in chickens. Three-week-old SPF chickens were intranasally inoculated with 10^6^ TCID_50_ of reHA_Δ227_ or reHA or received an equivalent volume of PBS (Mock). Three chickens per group were euthanized at each indicated time point for sample collection. **(A, B)** Concentrations of interferon-γ (IFN-γ) (A) and interleukin-10 (IL-10) (B) in serum. **(C–F)** Concentrations of IFN-γ **(C)**, IL-10 **(D)**, interleukin-1β (IL-1β) **(E)**, and monocyte chemoattractant protein-1 (MCP-1) (F) in lung homogenates. Cytokine and chemokine concentrations were determined by ELISA. Samples analyzed at 5 dpi were obtained from the same chickens used for viral RNA quantification and histopathological assessment in Fig 8. Data are presented as the mean ± SD (n = 3 per group per time point). dpi, days post-inoculation.

## Discussion

HA, the major surface glycoprotein of IAVs, undergoes rapid evolution driven by host immune pressure and environmental constraints. Previous functional studies of IAV HA have predominantly focused on the pleiotropic effects conferred by amino acid substitutions at key residues, particularly on the interconnected phenotypes of receptor-binding avidity, antigenicity, and pathogenicity. For instance, well-characterized substitutions at residues 226 and 228 within the RBD are known to modulate these adaptive traits [7,28,29]. While studies indicate that variation at HA residue 227 can modulate receptor-binding preference and avidity, potentially affecting host adaptation and tissue tropism [18,30], the phenotypic consequences of a deletion at this site have remained poorly understood. In this study, we elucidate the multifaceted consequences of a rare deletion at HA residue 227 (HA_Δ227_) in an H9N2 AIV strain. Through comparison of isogenic viruses, we found that the HA_Δ227_ was associated with multiple phenotypic changes consistent with a viral phenotypic trade-off between replicative capacity and disease severity: reduced replicative fitness accompanied by altered antigenicity and greater disease severity.

Viral infectivity is influenced by multiple factors, with protein stability being a key determinant. In the IAV life cycle, the HA precursor (HA0) is cleaved by host proteases into HA1—which contains the RBD essential for host cell attachment—and HA2, which harbors the fusion peptide. Under acidic conditions within the endosome, conformational changes in HA2 mediate fusion between the viral and endosomal membranes [24]. HA proteins with greater acid stability can maintain their prefusion conformation over a wider pH range, enabling efficient membrane fusion at the optimal pH [24,26,31]. Here, we found that reHA_Δ227_ lost hemagglutination activity more rapidly than reHA under gradually acidifying conditions, indicating that deletion of residue 227 increases HA sensitivity to acidic environments, which was further supported by the syncytium formation assay. Conversely, reHA_Δ227_ exhibited enhanced thermostability relative to reHA at both 37 °C and 42 °C, a trait that may promote viral survival under elevated temperatures. These divergent phenotypic outcomes highlight the dual regulatory role of residue 227 in environmental adaptation: its deletion reduces acid stability while concurrently enhancing thermal tolerance, reflecting a stability trade-off fine-tuned by this residue.

Structural analysis revealed that the HA_Δ227_ induced a subtle inward retraction of the 220-loop, thereby remodeling the local architecture of the receptor-binding site (RBS). Consistent with this structural change, reHA_Δ227_ exhibited increased receptor-binding avidity. Unexpectedly, this increase was accompanied by lower apparent virion-associated NA activity. Because reHA_Δ227_ and reHA contained the same NA segment derived from the same parental strain and consequently encoded identical NA proteins, the observed difference is unlikely to reflect altered intrinsic catalytic properties of NA. Instead, it may arise indirectly from the HA_Δ227_ or from other virion-level properties that influence the measurement of NA-associated activity. The opposing changes in HA receptor-binding avidity and apparent virion-associated NA activity raised the possibility that the deletion perturbed HA/NA functional balance, a key determinant of IAV fitness [9,27,32,33]. We examined this possibility using complementary molecular- and cellular-level readouts. The molecular-level assessment integrated the negative logarithm of the equilibrium dissociation constant (p*K*_D_), derived from the solid-phase binding assay, with the apparent NA catalytic efficiency (*V*_max_/*K*_m_), determined using the MUNANA assay. The cellular-level assessment integrated the amount of cell-bound infectious virus with the erythrocyte half-elution time. With the G1-lineage H9N2 virus serving as a reference benchmark, reHA exhibited a relationship between the selected HA- and NA-associated readouts broadly comparable to that of G1, whereas reHA_Δ227_ deviated markedly from both viruses (Fig 10A–10C). Together, these molecular- and cellular-level observations suggest that the HA_Δ227_ is associated with perturbed HA/NA functional balance.

**Fig 10:**
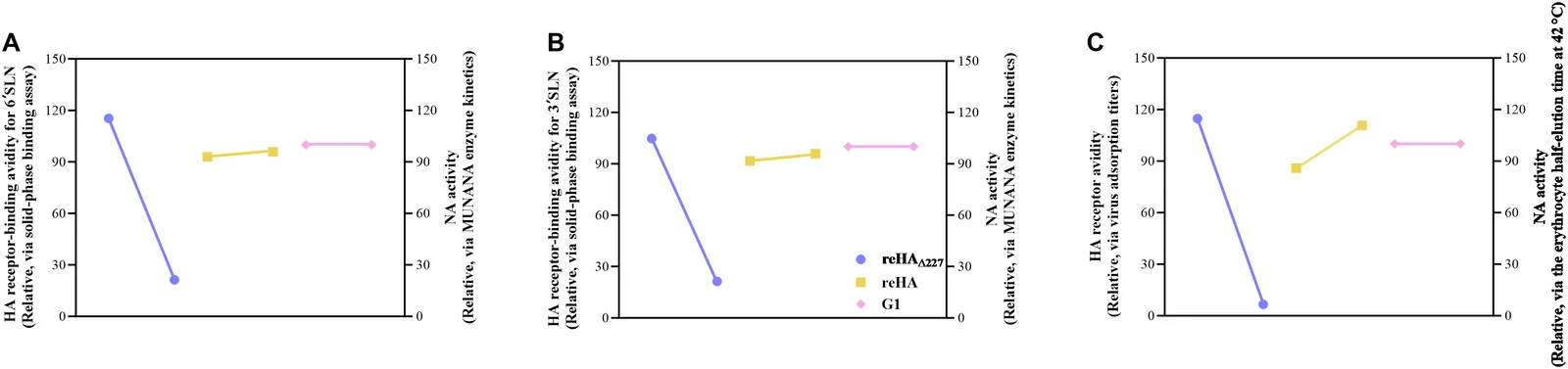
Comparative molecular and cellular profiling of HA/NA functional balance in H9N2 viruses. All reHA_Δ227_ and reHA values are expressed relative to the corresponding G1 value, which is set to 100%. (A, B) Molecular‑level assessment of HA/NA balance. HA‑receptor avidity toward 6′SLN (A) or 3′SLN (B) was expressed as p*K*_D_ [−log_10_(*K*_D_/1 M)]in solid-phase binding assays. In both panels, NA enzymatic activity was expressed as the catalytic efficiency (*V*_max_/*K*_m_), derived from Michaelis–Menten kinetics using the fluorogenic substrate 4-MUNANA. (C) Cellular‑level assessment of HA/NA balance. HA receptor-binding avidity (left) was quantified as the viral titer bound to the cell membrane in adsorption assays; NA-associated receptor-release kinetics (right) was expressed as the erythrocyte half‑elution time at 42 °C; a longer half-elution time indicates slower receptor release.

This apparent HA/NA functional imbalance may contribute to the replication deficit of reHA_Δ227_ and partly explain the rarity of this variant in surveillance datasets. Nevertheless, the HA_Δ227_ virus exhibited additional phenotypic changes of potential biological significance. First, the deletion increased the pH threshold for HA-mediated membrane fusion by 0.4 pH units. A higher fusion pH, indicative of reduced HA acid stability, has been correlated with increased pathogenicity in avian and mammalian models [8,34,35] and may therefore contribute to disease severity independently of a high viral burden. Notably, a previously reported H9N2 strain carrying the same HA_Δ227_ caused severe disease in chickens [14,36]. This observation suggests that, despite its apparent replicative disadvantage, the deletion variant can emerge and cause substantial outbreaks under permissive conditions. Its occurrence in vaccinated flocks, together with the altered antigenic profile observed in this study, raises the possibility that altered antigenicity may partly offset its reduced replicative fitness. Second, the deletion drove markedly altered antigenic reactivity (4.32 units), exceeding the established threshold of 2.0 for significant antigenic divergence [37]. The modeled conformational shift in the 220-loop, which overlaps a major antigenic epitope [5,8,17,18], may contribute to the observed reduction in cross-reactivity. Notably, the HA_Δ227_ virus was isolated from vaccinated chickens, although whether vaccine-induced immune pressure contributed to the emergence or maintenance of this variant remains unknown.

The principal finding of this study is that deletion of HA residue 227 uncoupled viral RNA burden from disease severity. Relative to the isogenic reHA virus, reHA_Δ227_ produced lower viral RNA levels in respiratory and cloacal swabs and in lung and duodenal tissues, yet caused more severe clinical disease, impaired weight gain, and greater pulmonary and duodenal injury. Viral RNA burden and tissue damage are related but biologically distinct dimensions of influenza pathogenesis and need not change in parallel [38,39]. Accordingly, the more severe phenotype of reHA_Δ227_ cannot be attributed to increased viral RNA burden. The inflammatory profile provides a plausible biological context for this discordance. Sustained increases in pulmonary IFN-γ and IL-1β are consistent with enhanced type II interferon signaling and inflammatory activation, respectively. Although both pathways contribute to antiviral defense, their excessive or persistent activation can intensify collateral tissue injury [40,41]. In parallel, the marked increase in MCP-1 is compatible with a pulmonary chemotactic environment that favors CCR2-dependent recruitment of inflammatory monocytes and their differentiation into monocyte-derived macrophages and dendritic cells [42]. Such cellular recruitment may contribute to interstitial inflammation even when the measured viral RNA burden is comparatively low. The concurrent rise in IL-10 is best interpreted cautiously. Rather than indicating enhanced inflammation per se, it may represent a compensatory counter-regulatory response to ongoing tissue injury [43]. Its increase alongside IFN-γ, IL-1β, and MCP-1 is consistent with the concurrent activation of pro-inflammatory and counter-regulatory responses; however, their temporal and functional relationships cannot be determined from the present data. Taken together, the coordinated mediator profile and more severe pathological phenotype are consistent with altered inflammatory regulation following reHA_Δ227_ infection. These data nevertheless establish association, not causation. Measurements in serum and whole-lung homogenates do not identify the cellular sources or spatial distribution of the mediators, nor do they distinguish protective antiviral signaling from responses that contribute to collateral injury. RT–qPCR further measures viral RNA rather than infectious virus and therefore cannot resolve differences in infectivity, tissue tropism, antigen persistence, or transient replication between sampling intervals. Definitive mechanistic attribution will require infectious-virus titration, cell-resolved immune profiling, spatial colocalization of viral antigen with inflammatory infiltrates, and targeted perturbation of candidate pathways. Despite these limitations, concordant clinical scores, weight trajectories, blinded histopathological assessments, and pulmonary mediator profiles suggest that HA_Δ227_ alters the nature of the host–pathogen interaction without increasing viral RNA burden. Detection of viral RNA in direct-contact birds further shows that both viruses retained the capacity to spread under the co-housing conditions used here; however, the present design does not establish equivalent transmission efficiency, which would require adequately powered analysis of transmission frequency, timing, and secondary shedding. More broadly, these findings underscore the need to evaluate naturally occurring H9N2 variants across integrated virological, clinical, pathological, and immunological endpoints. A lower viral RNA burden should not, by itself, be interpreted as evidence of reduced pathogenicity when host-associated tissue injury is disproportionately increased.

Overall, the HA_Δ227_ variant exemplifies a phenotypic trade-off in which reduced replicative fitness is accompanied by altered antigenicity, altered inflammatory responses, and greater tissue injury. This replication–disease trade-off has significant implications for influenza risk assessment. Traditional surveillance heavily prioritizes virological traits such as replication efficiency and genetic drift. These findings suggest that host pathological and inflammatory outcomes may provide complementary information to conventional virological characteristics when evaluating influenza virus variants, including those with reduced replicative fitness. In summary, characterization of the HA_Δ227_ provides insight into how specific genetic changes may alter the relationship between viral replication and disease severity, supporting the inclusion of pathological and inflammatory outcomes alongside conventional virological measurements in influenza surveillance and risk assessment.

## Materials and methods

### Ethics statement

All animal experiments were approved by the Animal Ethics Committee of the Jilin Academy of Agricultural Sciences (Protocol No. JNK20240108-2 and JNK20260709-1). Three-week-old SPF chickens (Beijing Boehringer Ingelheim Vital Biotechnology Co., Ltd., China) were maintained under standard husbandry conditions with ad libitum access to food and water.

### Phylogenetic analysis

The HA and NA gene sequences of H9N2 AIV strains isolated in China were retrieved from the Global Initiative on Sharing All Influenza Data (GISAID) database (https://www.gisaid.org/). Multiple sequence alignments were performed using MAFFT v7.520 on the online server (https://mafft.cbrc.jp/alignment/server/). Maximum-likelihood phylogenetic trees were constructed with IQ-TREE v3.0.1, implementing the best-fit substitution model determined by ModelFinder. Branch support was assessed with 1,000 ultra-fast bootstrap replicates. Branch lengths were scaled to nucleotide substitutions per site. For enhanced clarity, a simplified tree was generated by proportional and random subsampling of clades while preserving the original topological relationships.

### Cells

The cell lines used in this study included: human embryonic kidney epithelial cells (HEK 293T; ATCC CRL-11268), human lung adenocarcinoma cells (A549; ATCC CCL-18), African green monkey kidney epithelial cells (Vero; ATCC CCL-81), Madin-Darby canine kidney epithelial cells (MDCK; ATCC CCL-34), and primary chicken embryo fibroblasts (CEFs). Primary CEFs were prepared from 9- to 11-day-old specific pathogen-free (SPF) chicken embryos (Beijing Boehringer Ingelheim Vital Biotechnology Co., Ltd., China) as previously described [44]. All cells were maintained in Dulbecco’s modified Eagle medium (DMEM; HyClone) supplemented with 10% (v/v) fetal bovine serum (FBS; Gibco) and 1% (v/v) penicillin-streptomycin (Gibco) at 37 °C in a humidified incubator containing 5% CO_2_.

### Molecular modeling and HA structural analysis

The HA sequence of the H9N2 virus isolate A/chicken/Jilin/WJ1/2022, harboring the HA_Δ227_, was aligned with the publicly available H9N2 HA crystal structure (PDB ID: 1JSD, A/Swine/Hong Kong/9/98). Three-dimensional structures of the HA_Δ227_ variant and its methionine-restored (Met227) counterpart, excluding the transmembrane and cytoplasmic domains, were predicted using AlphaFold3 (https://alphafoldserver.com/). The predicted models and the 1JSD template were superimposed and visualized in PyMOL (v3.1.6.1; Schrödinger, LLC). Structural alignment was performed by iterative optimization using the align command with outlier cycle rejection [45]. Root-mean-square deviation (RMSD) values for Cα atoms of the entire globular head domain and of residues surrounding the deletion site were calculated to quantify conformational differences. The SA receptor analogs 3′SLN (BIRD ID: PRD_900067) and 6′SLN (BIRD ID: PRD_900046) were individually docked into the HA RBS of the HA_Δ227_ and Met227 models using AutoDock Vina v1.2.0 [46]. For each HA–receptor combination, a global search of ligand conformations within a grid box centered on the RBS was performed, followed by local optimization. Docking poses were ranked using the AutoDock Vina scoring function, and the 20 highest-ranked conformations for each complex were retained. The lowest-energy pose for each HA–receptor complex was selected for analysis of intermolecular interactions.

### Reverse genetics and virus rescue

The eight gene segments of A/chicken/Jilin/WJ1/2022 (H9N2) were used as templates for virus rescue via a bidirectional reverse genetics system. To generate the HA_Δ227_ mutant, the full-length cDNA of segment 4 (HA gene) was cloned into the pHW2000 plasmid. The methionine-restored (Met227) variant was constructed by site-directed mutagenesis. All eight plasmids were co-transfected into confluent HEK 293T cells using Lipofectamine 3000 (Invitrogen) [47]. At 48–72 h post-transfection, supernatants were harvested and inoculated into the allantoic cavity of 9- to 11-day-old SPF embryonated chicken eggs. Virus presence was confirmed by hemagglutination assay using 0.5% (v/v) chicken RBCs. Viral RNA was extracted from hemagglutination-positive allantoic fluid and reverse-transcribed, after which the complete viral genome was verified by Sanger sequencing.

### Virus titration

Viral titers were quantified using an endpoint dilution assay on monolayers of MDCK cells. Briefly, confluent cells in 96-well plates were inoculated with 100 μL of tenfold serial virus dilutions (five replicates per dilution) for 1 h at 37 °C. The inoculum was removed, and cells were overlaid with MEM containing 1 μg/mL TPCK-treated trypsin and 1% (v/v) FBS. After 18–20 h of incubation, cells were fixed with 4% (v/v) paraformaldehyde (30 min, 4 °C), permeabilized with 0.5% (v/v) Triton X-100, and blocked with 5% (w/v) skim milk (1 h, 37 °C). Viral antigen was detected by incubation with a mouse anti-influenza A virus (IAV) nucleoprotein (NP) monoclonal antibody (1:5,000; Santa Cruz), followed by an HRP-conjugated goat anti-mouse IgG secondary antibody (1:5,000; Abmart, China). Immunostained foci were visualized using the AEC chromogen substrate kit (Abcam) under an Olympus CKX53 microscope. Wells containing one or more NP-positive cells were scored as virus-positive, and TCID_50_ values were calculated using the Reed–Muench method [48].

### Acid stability assay

Virus suspensions (50 μL) were mixed with an equal volume of 100 mM prewarmed buffers adjusted to different pH values: sodium acetate (pH 4.0, 5.0), sodium phosphate (pH 6.0), or neutral phosphate-buffered saline (PBS; pH 7.0). Mixtures were incubated at 37 °C for 10 min and were then immediately cooled on ice. Residual hemagglutination activity was then measured using a standard hemagglutination assay [49] to assess viral acid stability, with changes in hemagglutination titer indicating the level of acid-induced inactivation.

### Thermostability assay

Aliquots (50 μL) of virus suspended in PBS were incubated at 37 °C or 42 °C for 0–120 h. At each time point, the samples were immediately placed on ice for 10 min. Residual hemagglutination activity was then measured using a hemagglutination assay to evaluate viral thermal stability.

### Syncytium formation assay

The pH threshold for HA-mediated membrane fusion was determined as described previously [50]. Briefly, Vero cells grown in 96-well cell culture plates were infected with the indicated viruses at an MOI of 3. After 16–20 hpi, the cells were washed with PBS and incubated with serum-free DMEM containing 5 μg/mL TPCK-treated trypsin for 15 min at 37 °C to cleave HA0. The medium was then replaced with prewarmed PBS adjusted to pH 5.0–6.1 at 0.1-unit intervals, and the cells were incubated for 15 min at 37 °C to trigger fusion. The fusion buffer was subsequently replaced with complete medium, and the cells were incubated for an additional 3 h before fixation with 4% paraformaldehyde. Syncytium formation was assessed by light microscopy in five randomly selected fields per condition. A condition was considered fusion-positive if at least one fused cell containing five or more nuclei was identified among the five fields examined. The fusion pH threshold was defined as the highest pH that yielded a fusion-positive result in all three independent experiments.

### Solid-phase binding assay

The relative receptor-binding avidity of viral HA for α2,3- and α2,6-linked sialosides was quantified using a streptavidin-biotin solid-phase binding assay [18]. Biotinylated polyacrylamide-conjugated glycans, 3′SLN and 6′SLN (GlycoNZ), were serially diluted and immobilized on streptavidin-coated 96-well plates (Thermo Fisher) overnight at 4 °C. The plates were blocked with 3% (w/v) BSA in PBST for 2 h at 37 °C. Virus preparations (32 HAU) were preincubated with 1 μM zanamivir (MedChemExpress) for 30 min at 37 °C to inhibit NA activity and then added to the glycan-coated wells for overnight incubation at 4 °C. Bound virus was detected using a mouse monoclonal antibody against IAV matrix protein 1 (M1; 1:2,000; Thermo Fisher), followed by HRP-conjugated goat anti-mouse IgG (1:5,000; Abmart) and TMB substrate. Absorbance was measured at 450 nm. The apparent dissociation constants (*K*_D_) were determined by Scatchard analysis using GraphPad Prism v10.1. Because the assay measures multivalent interactions between intact virions and surface-immobilized glycans, the fitted *K*_D_ values were interpreted as measures of apparent receptor-binding avidity rather than monovalent binding affinity.

### Hemagglutination assay

Virus samples were standardized to an initial hemagglutination titer of 256 HAU in PBS and subjected to twofold serial dilution in V-bottom 96-well plates. An equal volume of 0.5% (v/v) chicken RBCs in PBS was added to each well. The plates were gently mixed and incubated on ice for 1 h to allow hemagglutination while minimizing, but not necessarily eliminating, NA-mediated receptor cleavage and erythrocyte elution. The residual hemagglutination titer was defined as the reciprocal of the highest virus dilution that maintained a uniform sheet of agglutinated RBCs after incubation. Because all viruses were standardized to the same initial titer, this assay was used to compare their apparent capacity of intact virions to maintain HA-mediated erythrocyte agglutination under the specified conditions, rather than to measure the intrinsic receptor-binding avidity of HA independently of NA.

### Immunofluorescence assay

Viruses at an MOI of 10 were pre-incubated with zanamivir at 10 × IC₅₀ for 20 min at 37 °C. The virus–zanamivir mixtures were then cooled and added to A549 cells grown on glass coverslips. Viral adsorption was performed in serum-free MEM without TPCK-treated trypsin at 4 °C for 2 h. The cells were subsequently fixed with 4% paraformaldehyde, permeabilized with 0.1% (v/v) Triton X-100, and blocked with 2% (w/v) BSA. Cells were incubated overnight at 4 °C with a mouse monoclonal antibody against IAV NP (1:500; Santa Cruz), followed by DyLight 594-conjugated goat anti-mouse IgG (1:500; Earthox) for 1 h at room temperature (RT). Nuclei were counterstained with DAPI (1:2,000; Sigma) for 15 min at RT in the dark. Images were acquired using a fluorescence microscope (Olympus, Japan).

### Neuraminidase inhibition assay

Virus suspensions containing 10^4^ TCID_50_ in 100 μL of PBS were incubated with the indicated concentrations of zanamivir at 37 °C for 20 min. The NA activity of the virus-inhibitor mixture was then measured by adding 50 μM of the NA substrate 2′-(4-methylumbelliferyl)-α-D-N-acetylneuraminic acid (4-MUNANA; MedChemExpress). The resulting fluorescence of the product, 4-methylumbelliferone (4-MU) (Sigma-Aldrich), was measured immediately using a microplate reader (PerkinElmer) with excitation at 365 nm and emission at 450 nm. The half-maximal IC_50_ of zanamivir was determined to quantify its inhibitory effect on NA activity.

### Virus adsorption assay

Viruses at an MOI of 0.1 were pre-incubated with zanamivir at 10 × IC₅₀ for 20 min at 37 °C. The virus–zanamivir mixtures were then cooled, added to MDCK cells, and allowed to adsorb at 4 °C for 2 h. The supernatants were collected, and the cells were washed three times with ice-cold PBS to remove unbound virus. Ice-cold MEM was then added, and the cells were lysed by three freeze–thaw cycles at −80 °C. Viral titers in the supernatants and cell lysates were determined using the Reed–Muench method. The relative adsorption rate was calculated as the cell-associated viral titer divided by the total recovered viral titer (cell-associated plus unbound virus).

### Virus concentration and NA protein quantification

Virus-positive allantoic fluid harvested from embryonated chicken eggs was supplemented with PEG 6000 to a final concentration of 5% (w/v). The mixture was gently inverted three times at 20-min intervals and incubated overnight. The following day, samples were centrifuged at 12,000 × *g* for 20 min at 4 °C, and the resulting precipitate was collected. After removal of the supernatant, the precipitate was washed once with 2% (w/v) PEG 6000, centrifuged again, and resuspended in PBS [51]. The concentrated virus preparations were mixed with an appropriate volume of 5 × loading buffer, heat-denatured, and separated by SDS–PAGE. Proteins were subsequently transferred to PVDF membranes, blocked, and sequentially incubated with a rabbit polyclonal antibody against the NA protein of influenza A/H9N2 virus A/Hong Kong/1073/99 (1:2,000; Thermo Fisher Scientific, USA) and a goat anti-rabbit secondary antibody (1:5,000; Abmart, China). NA band intensities were quantified using ImageJ and normalized to that of the G1 strain, which was assigned a value of 1.0. Three independent experiments were performed to assess reproducibility.

### MUNANA-based NA enzyme kinetics

A standard curve for quantification of 4-MU was generated using 4-MU concentrations ranging from 0 to 400 μM in a black 96-well microplate. Fluorescence was monitored at 37 °C at 60-s intervals for 1 h using excitation and emission wavelengths of 365 and 450 nm, respectively. Fluorescence values recorded at 60 min were plotted against the corresponding 4-MU concentrations to generate the standard curve, which was subsequently used to convert fluorescence intensity into the amount of 4-MU released. For kinetic analysis, virus samples containing equivalent amounts of NA protein were prepared in 50 μL of reaction buffer (32.5 mM MES, 4 mM CaCl_2_, pH 6.5). Reactions were initiated by adding 50 μL of serially diluted 4-MUNANA to yield final substrate concentrations ranging from 0 to 400 μM. Fluorescence generated through NA-mediated hydrolysis of 4-MUNANA was monitored at 37 °C at 60-s intervals for 1 h. Fluorescence values recorded at 60 min were converted to the corresponding amounts of released 4-MU using the standard curve. Apparent reaction velocities were calculated from the amount of 4-MU released over the 60-min reaction period and plotted against substrate concentration. Michaelis–Menten kinetic parameters, including the maximum reaction velocity (*V*_max_) and Michaelis constant (*K*_m_), were determined by nonlinear regression using GraphPad Prism v10.1 [52].

### Enzyme-linked lectin assay (ELLA)

NA activity toward multivalent substrates was assessed by ELLA as described [53]. Briefly, 96-well plates were coated with 25 μg/mL fetuin (MedChemExpress) overnight at 4 °C. After blocking, virus samples containing equivalent amounts of NA protein were added and incubated for 18 h at 37 °C to allow desialylation. Plates were washed and incubated with 10 μg/mL biotinylated Sambucus nigra agglutinin (SNA, for α2,6-linked sialic acid) or Maackia amurensis lectin I (MAL I, for α2,3-linked sialic acid) (Vector Laboratories) for 2 h at 37 °C. Binding was detected with HRP-conjugated streptavidin (1:1,000; Invitrogen) and TMB substrate. Absorbance at 450 nm was inversely correlated with NA activity.

### Erythrocyte elution assay

Virus suspensions with equivalent hemagglutination titers were subjected to twofold serial dilution and mixed with an equal volume of 0.5% chicken RBCs in V-bottom 96-well plates. The mixtures were incubated at 4 °C for 1 h to allow adsorption. Plates were then shifted to 33 °C, 37 °C, or 42 °C. Hemagglutination patterns were recorded at 15-min intervals for the first hour and then every 30 min for up to 6 h. Elution kinetics were visualized by plotting hemagglutination titers against incubation time [9].

### Viral growth kinetics

MDCK or CEF cell monolayers were infected with virus at an MOI of 0.1 or 0.01. After 1 h adsorption at 37 °C, the inoculum was removed, cells were washed, and fresh infection medium (MEM with 2 μg/mL TPCK-treated trypsin) was added. Supernatants were harvested at specified time points. Viral titers in the supernatants were quantified and expressed as TCID_50_.

### Assessment of HA/NA functional balance

Because no established biochemical metric is available for quantifying HA/NA functional balance, we compared HA- and NA-associated activities using two complementary sets of readouts. At the molecular level, the apparent *K*_D_ values obtained from the solid-phase binding assay were transformed to p*K*_D_ values, calculated as −log_10_(*K*_D_/1 M) after conversion of *K*_D_ to molar units. Thus, higher p*K*_D_ values indicate greater apparent HA-mediated receptor-binding avidity. NA catalytic efficiency was represented by *V*_max_/*K*_m_, determined using MUNANA-based enzyme kinetics. At the cellular level, HA-mediated attachment was assessed by incubating zanamivir-pretreated virus with MDCK cells at 4 °C for 2 h at an MOI of 0.1 in the absence of TPCK-treated trypsin, followed by quantification of cell-bound infectious virus using the Reed–Muench method. NA-associated release from HA-receptor interactions was assessed using the erythrocyte half-elution time at 42 °C. These paired readouts were used to visualize relative HA/NA functional balance and were not interpreted as a validated quantitative balance index.

### Antigenic mapping

Antisera were generated in three-week-old SPF chickens by intramuscular immunization with 100 μL of formalin-inactivated virus preparation containing the equivalent of 10^6^ TCID_50_ based on the pre-inactivation virus titer. Sera were collected 21 dpi, heat-inactivated, and treated with receptor-destroying enzyme (Sigma). Hemagglutination inhibition (HI) assays were performed as described [54]. Briefly, serial twofold dilutions of RDE-treated sera were mixed with 4 HAU of virus in 96-well V-bottom plates. After 30 min incubation at 37 °C, 1% chicken RBCs were added. The HI titer was the reciprocal of the highest serum dilution that completely inhibited hemagglutination. Antigenic cartography was generated according to the method of Smith et al. [37].

### Animal experiments

#### Experimental design and clinical monitoring.

Forty-eight three-week-old SPF chicken were randomly assigned to the reHA_Δ227_, reHA, and mock groups, which were housed separately to prevent cross-group transmission. Each virus group comprised 18 chickens, of which 12 were inoculated intranasally, without anesthesia, with 10^6^ TCID_50_ of reHA_Δ227_ or reHA in 100 μL of PBS. The remaining six naïve chickens in each virus group were co-housed with the corresponding inoculated birds to serve as direct-contact chickens. The mock group comprised 12 chickens that were intranasally administered 100 μL of PBS without anesthesia. In each virus group, three inoculated chickens were euthanized at 1, 3, 5, and 7 dpi, respectively, for terminal sample collection (n = 3 per group per time point). Mock chickens were euthanized according to the same schedule. All chickens were observed daily at a fixed time for clinical abnormalities, including changes in demeanor and activity, feather condition and posture, respiratory signs, nasal and ocular signs, feed and water intake, and fecal appearance. Each category was scored from 0 to 3, yielding a total daily clinical score of 0–18 for each chicken. To maintain a consistent set of animals throughout the observation period and avoid changes in group composition resulting from scheduled necropsies, longitudinal statistical analysis was restricted to the three inoculated chickens per virus group assigned *a priori* to the 7-dpi terminal cohort. Chickens assigned to earlier necropsy time points were clinically monitored while alive, but their scores were not included in the longitudinal analysis. The same three inoculated chickens, together with three contact chickens designated *a priori* in each virus group, were monitored longitudinally for body weight. Body weight was recorded daily from 0 dpi. The percentage change from baseline was calculated as [(*W*_*i*,*t*_ − *W*_*i*,0_)/*W*_*i*,0_] × 100%, where *W*_*i*,0_ and *W*_*i*,*t*_ denote the body weight of bird *i* at 0 dpi and at time *t*, respectively.

#### Sample collection.

Oropharyngeal and cloacal swabs were collected immediately before euthanasia from the inoculated chickens assigned to each terminal sampling time point at 1, 3, 5, and 7 dpi (n = 3 per group per time point). Swabs were collected longitudinally from all six direct-contact chickens in each virus group at the corresponding time points. Following swab collection, the inoculated and mock chickens assigned to each time point were deeply anesthetized with isoflurane and euthanized by exsanguination while anesthesia was maintained. Death was confirmed before lung and duodenal tissues were collected. Blood was also collected from the terminally sampled chickens, and serum and lung homogenates obtained at 1, 3, 5, and 7 dpi were used for cytokine and chemokine analyses. Samples used for viral RNA quantification, histopathological assessment, and cytokine and chemokine analyses at 5 dpi were obtained from the same three chickens in each group. Viral RNA was quantified by RT–qPCR targeting the IAV M1 gene and expressed as log_10_ RNA copies per milliliter of swab medium or per milligram of tissue, as appropriate. The following primers were used: forward, 5′-CTTCTAACCGAGGTCGAAACG-3′; and reverse, 5′-GGCATTTTGGACAAAGCGTCTA-3′.

#### Histopathological evaluation.

Lung and duodenal tissues collected at 5 dpi were fixed in 10% neutral-buffered formalin, processed routinely, embedded in paraffin, sectioned, and stained with hematoxylin and eosin (H&E). Images were anonymized and independently evaluated by two pathologists blinded to group allocation. Substantial scoring discrepancies were resolved by joint review and consensus before animal-level scores were calculated. For pulmonary evaluation, each whole-lung section was initially surveyed under a 10 × objective. Eight non-overlapping fields distributed throughout the section were then randomly selected and imaged to minimize field-selection bias. In each field, the proportion of evaluable pulmonary parenchyma affected by inflammatory-cell infiltration, interstitial thickening, air-capillary disruption, hemorrhage, edema, or necrosis was estimated. Tissue folds, tears, sectioning artifacts, large airways, cartilage, and empty background were excluded. Lesions were graded on a predefined four-point scale integrating extent and morphological severity: 0, no substantive lesion (<1% of evaluable parenchyma); 1, mild focal or oligofocal lesions involving 1%–10%, with mild inflammatory-cell infiltration and/or slight interstitial thickening but preserved air-capillary architecture; 2, moderate multifocal lesions involving >10%–30%, with prominent inflammatory-cell infiltration, marked interstitial thickening, and focal partial disruption of air-capillary architecture; and 3, severe coalescing or diffuse lesions involving >30%, with extensive inflammatory-cell infiltration and widespread distortion or loss of pulmonary architecture. A score of 3 was also assigned when unequivocal extravascular hemorrhage, edema, or necrosis involved ≥10% of the evaluable area. When lesion extent and morphological severity mapped to different categories, the lower score was assigned unless the criteria for severe hemorrhage, edema, or necrosis were met. The mean of the eight field scores represented the pulmonary histopathology score for each chicken, which served as the biological replicate.

For duodenal assessment, each section was initially surveyed using a 20 × objective. Six non-overlapping fields distributed throughout the section were randomly selected and imaged. Fields containing interpretable mucosal tissue were included regardless of whether intact villus–crypt units remained identifiable, because extensive villous or epithelial loss could represent genuine pathological injury. Higher magnification was used when necessary to confirm epithelial injury, inflammatory-cell infiltration, hemorrhage, edema, or necrosis. Tangentially sectioned villi, tissue folds, tears, and unequivocal processing artifacts were excluded; genuine villous shedding, epithelial sloughing, and mucosal destruction were retained as pathological findings. Duodenal lesions were graded on a predefined four-point semiquantitative scale incorporating lesion extent, inflammatory severity, mucosal architectural damage, and the presence of hemorrhage, edema, or necrosis. A score of 0 indicated no substantive lesion, affecting <1% of the evaluable tissue, with intact epithelium, normally arranged villi and crypts, and no abnormal inflammatory-cell infiltration. A score of 1 indicated mild, focal or oligofocal lesions affecting 1%–10% of the evaluable tissue, characterized by mild inflammatory-cell infiltration, slight expansion of the lamina propria, and occasional mild villous blunting or epithelial injury without crypt destruction. A score of 2 indicated moderate, multifocal lesions affecting >10%–30% of the evaluable tissue, with prominent inflammatory-cell infiltration, marked expansion of the lamina propria, multifocal villous shortening or fusion, focal epithelial erosion or sloughing, and/or focal crypt injury, while the overall mucosal architecture remained recognizable. A score of 3 indicated severe, coalescing, or diffuse lesions affecting >30% of the evaluable tissue, with widespread villous loss or fusion, extensive epithelial ulceration or sloughing, marked crypt destruction, or severe disruption of the normal mucosal architecture. A score of 3 was also assigned when unequivocal extravascular hemorrhage, edema, or necrosis affected ≥10% of the evaluable tissue or extended deeply into the intestinal wall. When extent and severity mapped to different categories, the lower category was assigned unless the criteria for severe hemorrhage, edema, necrosis, or deep mural injury were met. The mean of the six field scores represented the duodenal histopathology score for each chicken, which served as the biological replicate.

#### Cytokine and chemokine analysis.

Concentrations of interferon-γ (IFN-γ) and interleukin-10 (IL-10) in serum and lung homogenates at 1, 3, 5, and 7 dpi were measured using commercial ELISA kits (Invitrogen). Pulmonary interleukin-1β (IL-1β) and monocyte chemoattractant protein-1 (MCP-1) in lung homogenates at 1, 3, 5, and 7 dpi were quantified using specific ELISA kits (MyBioSource). Assays were performed according to the manufacturers’ instructions, and concentrations were expressed as pg/mL.

### Statistical analysis

Data are presented as the mean ± standard deviation (SD), unless otherwise indicated. For *in vitro* assays, data were obtained from at least three independent experiments. Sample sizes for animal experiments are specified in the corresponding figure legends. Differences between two groups were evaluated using an unpaired two-tailed Student’s *t*-test. Comparisons among multiple groups were performed by one-way or two-way ANOVA. All analyses were conducted using GraphPad Prism v10.1. Differences were considered not statistically significant when *p* > 0.05 (ns) and statistically significant when *p* < 0.05. Statistical significance is indicated as follows: *p < 0.05, **p < 0.01, ***p < 0.001, and ****p < 0.0001.

## Supporting information

S1 Graphical abstract The HA_Δ227_ disrupts HA/NA functional balance and restricts viral replication, yet paradoxically exacerbates pulmonary immunopathology through dysregulated host immune responses. These findings reveal an unexpected dissociation between viral replication and disease severity driven by the HA_Δ227_.
(TIF)

S1 Fig Inhibition of NA activity by zanamivir. NA activity was measured using the fluorescent substrate 2′-(4-methylumbelliferyl)-α-D-N-acetylneuraminic acid (4-MUNANA), and half-maximal inhibitory concentration (IC_50_) values were calculated by nonlinear regression. Data are presented as the mean ± SD of three independent experiments.
(TIF)

S2 Fig Relative NA protein levels in concentrated virion preparations. Virions concentrated using PEG 6000 were analyzed by western blotting with a polyclonal antibody against the NA protein of influenza A(H9N2) virus A/Hong Kong/1073/99 (Thermo Fisher Scientific, USA). (A–C) Results from three independent experiments. NA band intensities were quantified using ImageJ and normalized to that of the G1 strain, which was set to 1.0. Values below the bands indicate relative NA protein levels. **S2A Fig. Replicate 1.tif.** Western blot image showing relative NA protein levels in concentrated virion preparations from the first independent experiment.
(TIF)

S2B Fig Replicate 2.tif. Western blot image showing relative NA protein levels in concentrated virion preparations from the second independent experiment.
(TIF)

S2C Fig Replicate 3.tif. Western blot image showing relative NA protein levels in concentrated virion preparations from the third independent experiment.
(TIF)

S3 Fig Detection of influenza A virus NP in lung tissues of three-week-old SPF chickens. Representative immunofluorescence images of lung sections collected at 5 days post-inoculation (dpi) from three PBS-treated mock-control chickens (Mock 1–3) and three chickens inoculated with 10^6^ TCID_50_ of either reHA_Δ227_ (reHA_Δ227_ 1–3) or reHA (reHA 1–3). Viral NP immunoreactivity is shown in green (FITC), and nuclei are counterstained with DAPI (blue). Images were acquired using a 10 × objective. Scale bars, 50 μm.
(TIF)

S4 Fig Detection of influenza A virus NP in duodenal tissues of three-week-old SPF chickens. Representative immunofluorescence images of duodenal sections collected at 5 days post-inoculation (dpi) from three PBS-treated mock-control chickens (Mock 1–3) and three chickens inoculated with 10^6^ TCID_50_ of either reHA_Δ227_ (reHA_Δ227_ 1–3) or reHA (reHA 1–3). Viral NP immunoreactivity is shown in green (FITC), and nuclei are counterstained with DAPI (blue). Images were acquired using a 10 × objective. Scale bars, 50 μm.
(TIF)

S1 Table Selective-pressure analysis of amino acid sites within the 220-loop of the H9N2 virus HA receptor-binding domain. Site-specific selection was evaluated using three codon-based methods: Single-Likelihood Ancestor Counting (SLAC), Fast, Unconstrained Bayesian AppRoximation (FUBAR), and Fixed Effects Likelihood (FEL). Evidence of negative (purifying) selection was defined as follows: for SLAC, a negative *d*N − *d*S estimate with a one-sided *P* value ≤ 0.10; for FUBAR, a posterior probability of α > β ≥ 0.90, where α and β denote the synonymous and nonsynonymous substitution rates, respectively; and for FEL, an estimated β < α with a one-sided *P* value ≤ 0.10. Sites meeting the criterion for a given method were considered to show evidence of pervasive negative selection according to that method.
(DOCX)

S2 Table Apparent receptor-binding avidity of H9N2 viruses. Apparent dissociation constants (*K*_D_) for interactions between intact virions and sialylglycopolymer receptor analogs were derived from the solid-phase binding data. Raw absorbance values at 450 nm (*A*_450_) were plotted against receptor concentration (*C*), and the Scatchard relationship *A*/*C* = *A*_max_/*K*_D_ − *A*/*K*_D_ was used to estimate *K*_D_ from the slope (−1/*K*_D_). Lower apparent *K*_D_ values indicate greater apparent receptor-binding avidity [55]. Data are presented as the mean of three independent experiments.
(DOCX)

S3 Table Kinetic parameters of NA activity for H9N2 viruses. Data are presented as the mean ± SD of three independent experiments.
(DOCX)

S4 Table Cross-hemagglutination inhibition (HI) titers between H9N2 virus strains and antisera. Data are presented as the mean of three independent experiments.
(DOCX)

S5 Table Field-level histopathological scores and corresponding scoring rationales for lung tissues following H9N2 virus infection in three-week-old SPF chickens. Lung tissues were collected at 5 days post-inoculation (dpi) from three PBS-treated mock-control chickens (Mock 1–3) and three chickens inoculated with 10^6^ TCID_50_ of either reHA_Δ227_ (reHA_Δ227_ 1–3) or reHA (reHA 1–3). Eight randomly selected, non-overlapping fields from each H&E-stained lung section were evaluated using the predefined 0–3 histopathological scoring system described in the Methods. For each field, the assigned score and its corresponding morphological rationale are provided. The mean of the eight field-level scores was calculated as the overall pulmonary histopathological score for each chicken.
(XLSX)

S6 Table Field-level histopathological scores and corresponding scoring rationales for duodenal tissues following H9N2 virus infection in three-week-old SPF chickens. Duodenal tissues were collected at 5 days post-inoculation (dpi) from three PBS-treated mock-control chickens (Mock 1–3) and three chickens inoculated with 10^6^ TCID_50_ of either reHA_Δ227_ (reHA_Δ227_ 1–3) or reHA (reHA 1–3). Six randomly selected, non-overlapping fields from each H&E-stained duodenal section were evaluated using the predefined 0–3 histopathological scoring system described in the Methods. For each field, the assigned score and its corresponding morphological rationale are provided. The mean of the six field-level scores was calculated as the overall duodenal histopathological score for each chicken.
(XLSX)

S1 Rawgel Original, uncropped western blot images corresponding to S2 Fig. (PDF)

S1 Supporting information Contains supplementary figure legends, the supplementary table, and supporting information. (DOCX)
