# Supplementary material for: A single-residue deletion in H9N2 hemagglutinin uncouples viral burden from disease severity
Source: PLoS Pathog. 2026 Nov 5;22(11):e1014776. doi: 10.1371/journal.ppat.1014776 (PMC13759047; doi:10.1371/journal.ppat.1014776)

Fig.S2A

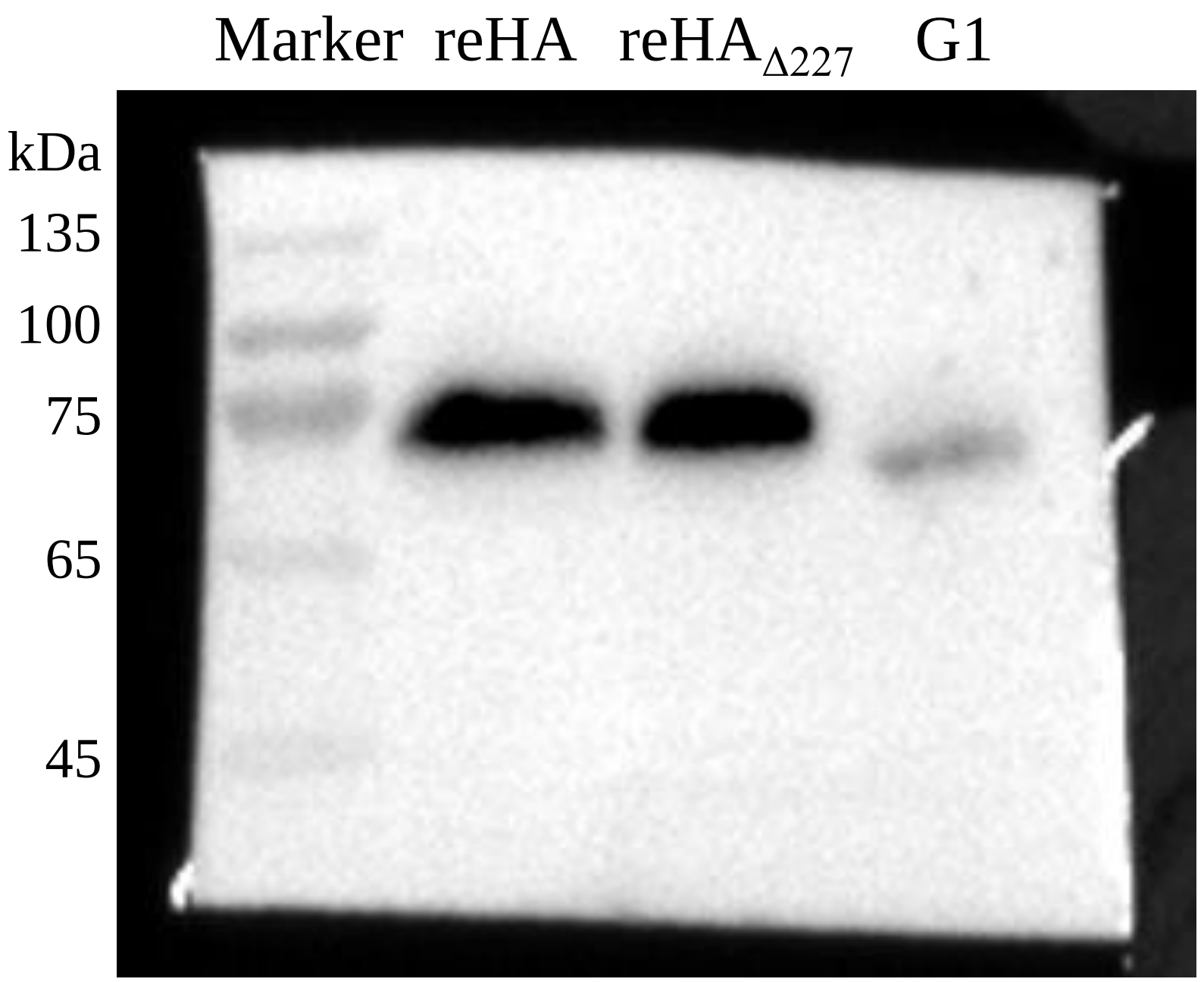

Fig.S2B

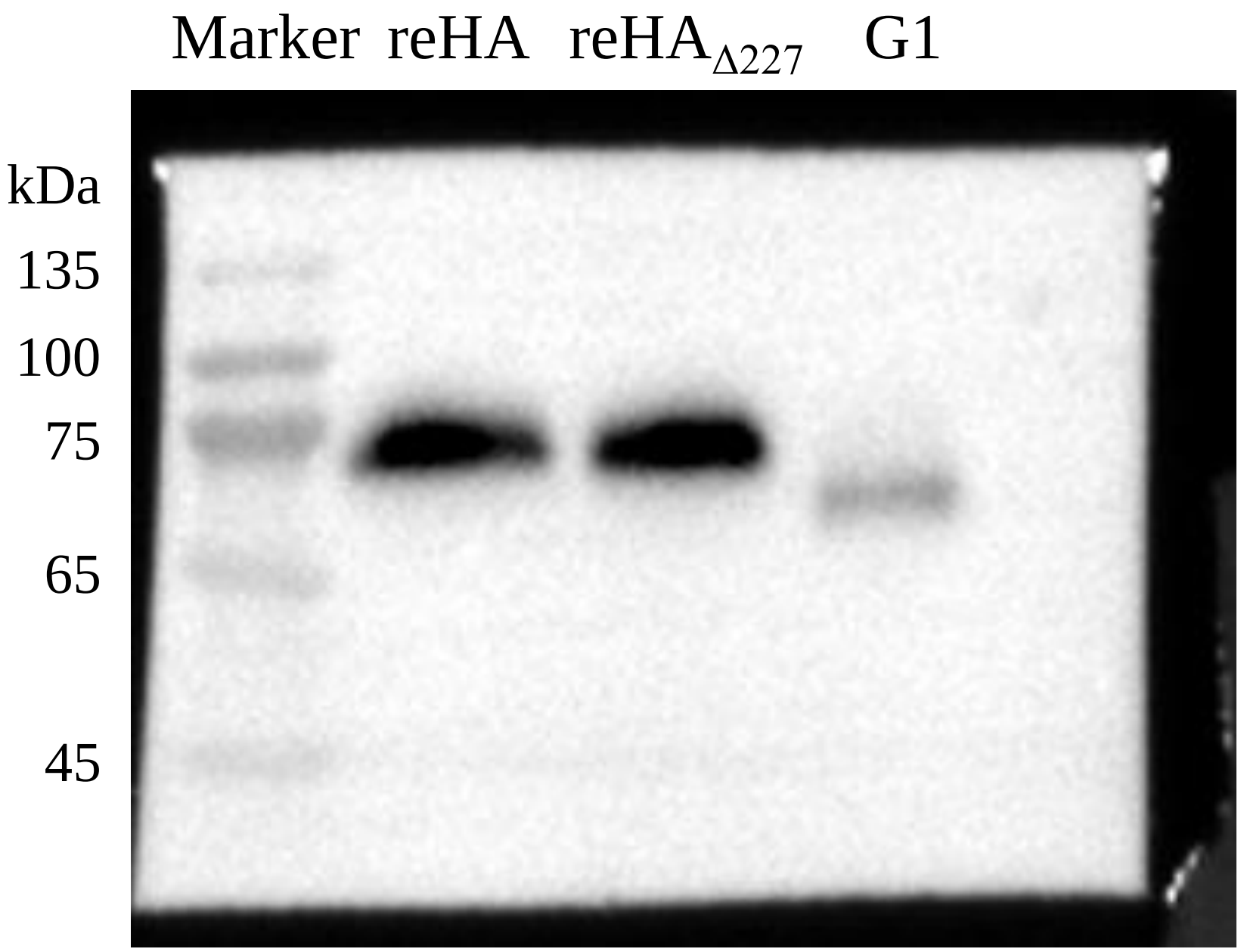

Fig.S2C

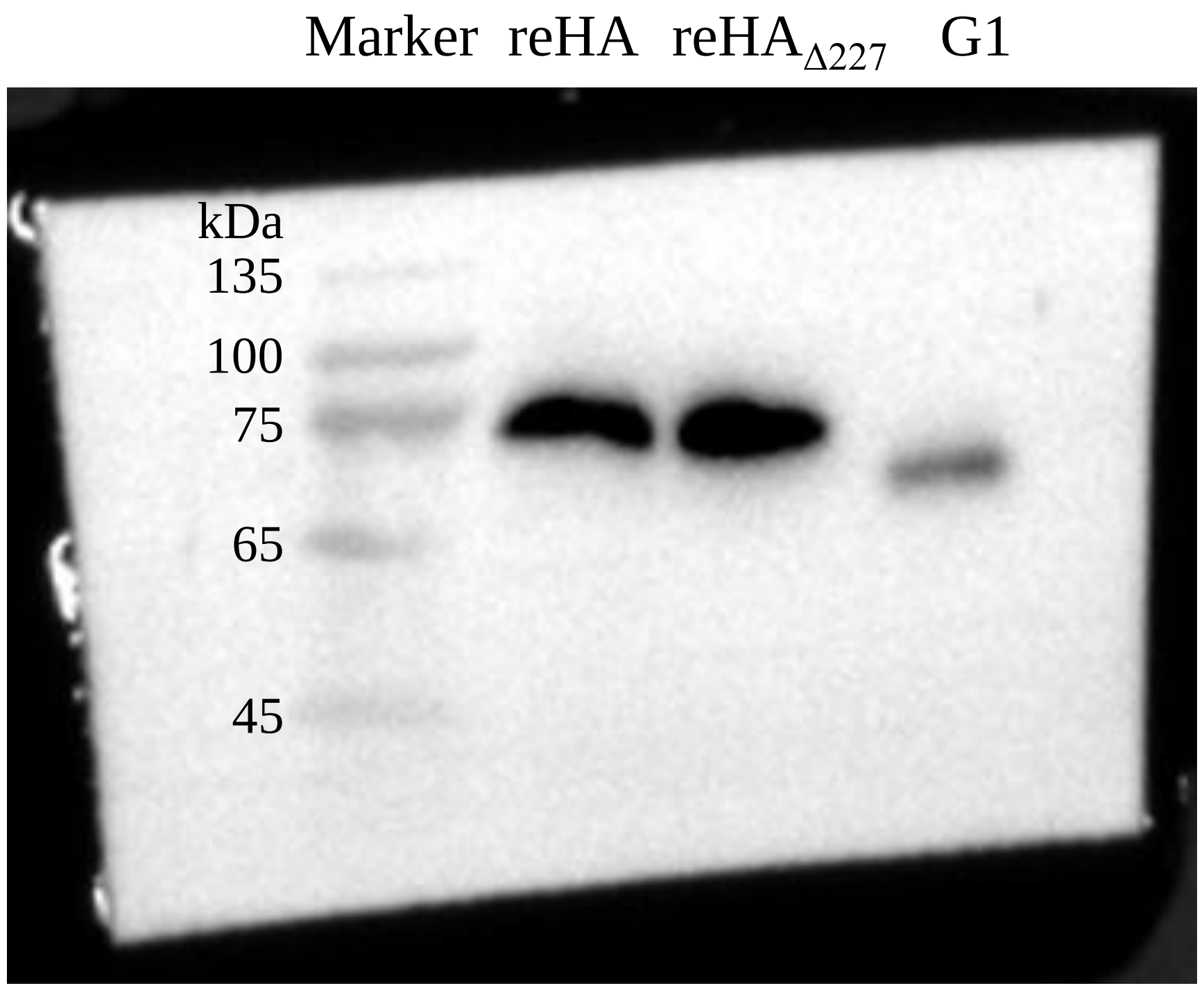

Supplement: Supplementary file 1 [file ppat.1014776.s014.pdf]
