# Supplementary material for: A single-residue deletion in H9N2 hemagglutinin uncouples viral burden from disease severity
Source: PLoS Pathog. 2026 Nov 5;22(11):e1014776. doi: 10.1371/journal.ppat.1014776 (PMC13759047; doi:10.1371/journal.ppat.1014776)
Supplement: Supplementary file 2 [file ppat.1014776.s008.docx]

**Table S1. Selective-pressure analysis of amino acid sites within the 220-loop of the H9N2 virus HA receptor-binding domain.** Site-specific selection was evaluated using three codon-based methods: Single-Likelihood Ancestor Counting (SLAC), Fast, Unconstrained Bayesian AppRoximation (FUBAR), and Fixed Effects Likelihood (FEL). Evidence of negative (purifying) selection was defined as follows: for SLAC, a negative *d*N − *d*S estimate with a one-sided *P* value ≤ 0.10; for FUBAR, a posterior probability of α > β ≥ 0.90, where α and β denote the synonymous and nonsynonymous substitution rates, respectively; and for FEL, an estimated β < α with a one-sided *P* value ≤ 0.10. Sites meeting the criterion for a given method were considered to show evidence of pervasive negative selection according to that method.

| Position | SLAC | |  | FUBAR | |  | FEL | | Methods supporting negative selection |
| --- | --- | --- | --- | --- | --- | --- | --- | --- | --- |
|  | dN - dS^1^ | P value^2^ |  | β - α^1^ | P(α > β)^3^ |  | P value^4^ | Classification^5^ |  |
| 220 | -1.870 | 0.05180000 |  | -1.652 | 0.992 |  | 0.0309 | Purifying | 3/3 |
| 221 | -1.780 | 0.02730000 |  | -1.870 | 0.992 |  | 0.0066 | Purifying | 3/3 |
| 222 | -0.276 | 0.45600000 |  | -0.956 | 0.768 |  | 0.4312 | Neutral | 0/3 |
| 223 | -4.650 | 0.00000023 |  | -2.570 | 1.000 |  | 0.0000 | Purifying | 3/3 |
| 224 | -4.120 | 0.00214000 |  | -1.632 | 0.910 |  | 0.0017 | Purifying | 3/3 |
| 225 | -3.400 | 0.00200000 |  | -2.684 | 0.999 |  | 0.0000 | Purifying | 3/3 |
| 226 | -2.770 | 0.00740000 |  | -1.710 | 0.993 |  | 0.0014 | Purifying | 3/3 |
| 227 | -4.770 | 0.03270000 |  | -1.968 | 0.948 |  | 0.0298 | Purifying | 3/3 |
| 228 | -0.973 | 0.23300000 |  | -1.441 | 0.890 |  | 0.0833 | Purifying | 1/3 |

^1^ Difference between the estimated nonsynonymous and synonymous substitution rates (*d*N − *d*S).

^2^ One-sided *P* value for the SLAC test of whether the nonsynonymous substitution rate is lower than the synonymous substitution rate.

^3^ Posterior probability that the synonymous substitution rate (α) exceeds the nonsynonymous substitution rate (β), consistent with negative selection.

^4^ *P* value from the FEL likelihood-ratio test comparing the nonsynonymous and synonymous substitution rates.

^5^ Site-level selection classification according to FEL based on the estimated substitution rates and the specified significance threshold.
