# Supplementary material for: A single-residue deletion in H9N2 hemagglutinin uncouples viral burden from disease severity
Source: PLoS Pathog. 2026 Nov 5;22(11):e1014776. doi: 10.1371/journal.ppat.1014776 (PMC13759047; doi:10.1371/journal.ppat.1014776)
Supplement: Supplementary file 3 [file ppat.1014776.s009.docx]

**Table S2. Apparent receptor-binding avidity of H9N2 viruses.** Apparent dissociation constants (*K*_D_) for interactions between intact virions and sialylglycopolymer receptor analogs were derived from the solid-phase binding data. Raw absorbance values at 450 nm (*A*_450_) were plotted against receptor concentration (*C*), and the Scatchard relationship *A*/*C* = *A*_max_/*K*_D_ − *A*/*K*_D_ was used to estimate *K*_D_ from the slope (−1/*K*_D_). Lower apparent *K*_D_ values indicate greater apparent receptor-binding avidity [1]. Data are presented as the mean of three independent experiments.

| Receptor | Virus | Apparent *K*_D_ (μg/mL) | *A*_max_ |
| --- | --- | --- | --- |
| 6′SLN | reHA_∆227_ | 0.30 | 1.21 |
|  | reHA | 0.38 | 0.87 |
|  | G1 | 0.36 | 1.02 |
| 3′SLN | reHA_∆227_ | 0.46 | 0.95 |
|  | reHA | 0.50 | 0.72 |
|  | G1 | 0.47 | 0.84 |

**Reference**

1.	Gambaryan AS, Matrosovich MN. A solid-phase enzyme-linked assay for influenza virus receptor-binding activity. J Virol Methods. 1992;39(1-2):111-23. Epub 1992/09/01. doi: 10.1016/0166-0934(92)90130-6. PubMed PMID: 1430058.
