# Supplementary material for: A single-residue deletion in H9N2 hemagglutinin uncouples viral burden from disease severity
Source: PLoS Pathog. 2026 Nov 5;22(11):e1014776. doi: 10.1371/journal.ppat.1014776 (PMC13759047; doi:10.1371/journal.ppat.1014776)
Supplement: Supplementary file 4 [file ppat.1014776.s010.docx]

**Table S3. Kinetic parameters of NA activity for H9N2 viruses.** Data are presented as the mean ± SD of three independent experiments.

| Virus | *V*_max_ (μM/min) | *K*_m_ (μM) | *V*_max_/*K*_m_ |
| --- | --- | --- | --- |
| reHA_∆227_ | 4.78 ± 0.16 | 154.05 ± 12.03 | 0.030 ± 0.003 |
| reHA | 11.26 ± 0.24 | 80.86 ± 4.84 | 0.140 ± 0.009 |
| G1 | 9.65 ± 0.26 | 66.39 ± 5.25 | 0.150 ± 0.012 |
