# Supplementary material for: A single-residue deletion in H9N2 hemagglutinin uncouples viral burden from disease severity
Source: PLoS Pathog. 2026 Nov 5;22(11):e1014776. doi: 10.1371/journal.ppat.1014776 (PMC13759047; doi:10.1371/journal.ppat.1014776)
Supplement: Supplementary file 5 [file ppat.1014776.s011.docx]

**Table S4. Cross-hemagglutination inhibition (HI) titers between H9N2 virus strains and antisera.** Data are presented as the mean of three independent experiments.

| Virus | HI titer of antiserum (log_2_) | |
| --- | --- | --- |
|  | reHA_∆227_ | reHA |
| reHA_∆227_ | 14 | 6 |
|  | 12 | 7 |
|  | 14 | 7 |
| reHA | 10 | 12 |
|  | 9 | 12 |
|  | 11 | 12 |
