# Supplementary material for: A single-residue deletion in H9N2 hemagglutinin uncouples viral burden from disease severity
Source: PLoS Pathog. 2026 Nov 5;22(11):e1014776. doi: 10.1371/journal.ppat.1014776 (PMC13759047; doi:10.1371/journal.ppat.1014776)
Supplement: Supplementary file 6 [file ppat.1014776.s015.docx]

**Supplementary Figure Legends**

**Fig. S1. Inhibition of NA activity by zanamivir.** NA activity was measured using the fluorescent substrate 2′-(4-methylumbelliferyl)-α-D-N-acetylneuraminic acid (4-MUNANA), and half-maximal inhibitory concentration (IC_50_) values were calculated by nonlinear regression. Data are presented as the mean ± SD of three independent experiments.

**Fig. S2. Relative NA protein levels in concentrated virion preparations.** Virions concentrated using PEG 6000 were analyzed by western blotting with a polyclonal antibody against the NA protein of influenza A(H9N2) virus A/Hong Kong/1073/99 (Thermo Fisher Scientific, USA). (A–C) Results from three independent experiments. NA band intensities were quantified using ImageJ and normalized to that of the G1 strain, which was set to 1.0. Values below the bands indicate relative NA protein levels.

**Fig. S3. Detection of influenza A virus NP in lung tissues of three-week-old SPF chickens.** Representative immunofluorescence images of lung sections collected at 5 days post-inoculation (dpi) from three PBS-treated mock-control chickens (Mock 1–3) and three chickens inoculated with 10^6^ TCID_50_ of either reHA_Δ227_ (reHA_Δ227_ 1–3) or reHA (reHA 1–3). Viral NP immunoreactivity is shown in green (FITC), and nuclei are counterstained with DAPI (blue). Images were acquired using a 10× objective. Scale bars, 50 μm.

**Fig. S4. Detection of influenza A virus NP in duodenal tissues of three-week-old SPF chickens.** Representative immunofluorescence images of duodenal sections collected at 5 days post-inoculation (dpi) from three PBS-treated mock-control chickens (Mock 1–3) and three chickens inoculated with 10^6^ TCID_50_ of either reHA_Δ227_ (reHA_Δ227_ 1–3) or reHA (reHA 1–3). Viral NP immunoreactivity is shown in green (FITC), and nuclei are counterstained with DAPI (blue). Images were acquired using a 10× objective. Scale bars, 50 μm.

**Supplementary Table**

**Table S1. Selective-pressure analysis of amino acid sites within the 220-loop of the H9N2 virus HA receptor-binding domain.** Site-specific selection was evaluated using three codon-based methods: Single-Likelihood Ancestor Counting (SLAC), Fast, Unconstrained Bayesian AppRoximation (FUBAR), and Fixed Effects Likelihood (FEL). Evidence of negative (purifying) selection was defined as follows: for SLAC, a negative *d*N − *d*S estimate with a one-sided *P* value ≤ 0.10; for FUBAR, a posterior probability of α > β ≥ 0.90, where α and β denote the synonymous and nonsynonymous substitution rates, respectively; and for FEL, an estimated β < α with a one-sided *P* value ≤ 0.10. Sites meeting the criterion for a given method were considered to show evidence of pervasive negative selection according to that method.

**Table S2. Apparent receptor-binding avidity of H9N2 viruses.** Apparent dissociation constants (*K*_D_) for interactions between intact virions and sialylglycopolymer receptor analogs were derived from the solid-phase binding data. Raw absorbance values at 450 nm (*A*_450_) were plotted against receptor concentration (*C*), and the Scatchard relationship *A*/*C* = *A*_max_/*K*_D_ − *A*/*K*_D_ was used to estimate *K*_D_ from the slope (−1/*K*_D_). Lower apparent *K*_D_ values indicate greater apparent receptor-binding avidity [1]. Data are presented as the mean of three independent experiments.

**Table S3. Kinetic parameters of NA activity for H9N2 viruses.** Data are presented as the mean ± SD of three independent experiments.

**Table S4. Cross-hemagglutination inhibition (HI) titers between H9N2 virus strains and antisera.** Data are presented as the mean of three independent experiments.

**Table S5. Field-level histopathological scores and corresponding scoring rationales for lung tissues following H9N2 virus** **infection in three-week-old SPF chickens.** Lung tissues were collected at 5 days post-inoculation (dpi) from three PBS-treated mock-control chickens (Mock 1–3) and three chickens inoculated with 10^6^ TCID_50_ of either reHA_Δ227_ (reHA_Δ227_ 1–3) or reHA (reHA 1–3). Eight randomly selected, non-overlapping fields from each H&E-stained lung section were evaluated using the predefined 0–3 histopathological scoring system described in the Methods. For each field, the assigned score and its corresponding morphological rationale are provided. The mean of the eight field-level scores was calculated as the overall pulmonary histopathological score for each chicken.

**Table S6. Field-level histopathological scores and corresponding scoring rationales for duodenal tissues following H9N2 virus infection in three-week-old SPF chickens.** Duodenal tissues were collected at 5 days post-inoculation (dpi) from three PBS-treated mock-control chickens (Mock 1–3) and three chickens inoculated with 10^6^ TCID_50_ of either reHA_Δ227_ (reHA_Δ227_ 1–3) or reHA (reHA 1–3). Six randomly selected, non-overlapping fields from each H&E-stained duodenal section were evaluated using the predefined 0–3 histopathological scoring system described in the Methods. For each field, the assigned score and its corresponding morphological rationale are provided. The mean of the six field-level scores was calculated as the overall duodenal histopathological score for each chicken.

**Supporting Information**

**S1_Rawgel.pdf.** Original, uncropped western blot images corresponding to Fig. S2.

**S1_Supporting Information.docx.** Contains supplementary figure legends, the supplementary table, and supporting information.

**Fig. S2A. Replicate 1.tif.** Western blot image showing relative NA protein levels in concentrated virion preparations from the first independent experiment.

**Fig. S2B. Replicate 2.tif.** Western blot image showing relative NA protein levels in concentrated virion preparations from the second independent experiment.

**Fig. S2C. Replicate 3.tif.** Western blot image showing relative NA protein levels in concentrated virion preparations from the third independent experiment.

**Reference**

1.	Gambaryan AS, Matrosovich MN. A solid-phase enzyme-linked assay for influenza virus receptor-binding activity. J Virol Methods. 1992;39(1-2):111-23. Epub 1992/09/01. doi: 10.1016/0166-0934(92)90130-6. PubMed PMID: 1430058.
